# Frequent alterations in cytoskeleton remodelling genes in primary and metastatic lung adenocarcinomas

**DOI:** 10.1038/ncomms10131

**Published:** 2015-12-09

**Authors:** Kui Wu, Xin Zhang, Fuqiang Li, Dakai Xiao, Yong Hou, Shida Zhu, Dongbing Liu, Xiaofei Ye, Mingzhi Ye, Jie Yang, Libin Shao, Hui Pan, Na Lu, Yuan Yu, Liping Liu, Jin Li, Liyan Huang, Hailing Tang, Qiuhua Deng, Yue Zheng, Lihua Peng, Geng Liu, Xia Gu, Ping He, Yingying Gu, Weixuan Lin, Huiming He, Guoyun Xie, Han Liang, Na An, Hui Wang, Manuel Teixeira, Joana Vieira, Wenhua Liang, Xin Zhao, Zhiyu Peng, Feng Mu, Xiuqing Zhang, Xun Xu, Huanming Yang, Karsten Kristiansen, Jian Wang, Nanshan Zhong, Jun Wang, Qiang Pan-Hammarström, Jianxing He

**Affiliations:** 1 BGI-Shenzhen, Beishan Industrial Zone, Yantian District, Shenzhen 518083, China; 2 Department of Thoracic Surgery, The First Affiliated Hospital of Guangzhou Medical University, Guangzhou 510120, China; 3 Guangzhou Institute of Respiratory Disease & State Key Laboratory of Respiratory Disease, Guangzhou 510120, China; 4 National Clinical Research Center for Respiratory Disease, Guangzhou 510120, China; 5 Department of Biology, University of Copenhagen, DK-2200 Copenhagen N, Denmark; 6 Research Center for Translational Medicine, The First Affiliated Hospital of Guangzhou Medical University, Guangzhou 510120, China; 7 Department of Laboratory of Medicine, Karolinska Institutet, Stockholm 14186, Sweden; 8 Guangzhou Key Laboratory of Cancer Trans-Omics Research, BGI-Guangzhou, Guangzhou 510006, China; 9 Department of Pathology, The First Affiliated Hospital of Guangzhou Medical University, Guangzhou 510120, China; 10 Genetics Department and Research Center, Portuguese Oncology Institute, Porto 4200-072, Portugal; 11 BGI-Wuhan, Wuhan 430075, China; 12 James D. Watson Institute of Genome Sciences, Zhejiang University, Hangzhou 310058, China

## Abstract

The landscape of genetic alterations in lung adenocarcinoma derived from Asian patients is largely uncharacterized. Here we present an integrated genomic and transcriptomic analysis of 335 primary lung adenocarcinomas and 35 corresponding lymph node metastases from Chinese patients. Altogether 13 significantly mutated genes are identified, including the most commonly mutated gene *TP53* and novel mutation targets such as *RHPN2*, *GLI3* and *MRC2*. *TP53* mutations are furthermore significantly enriched in tumours from patients harbouring metastases. Genes regulating cytoskeleton remodelling processes are also frequently altered, especially in metastatic samples, of which the high expression level of *IQGAP3* is identified as a marker for poor prognosis. Our study represents the first large-scale sequencing effort on lung adenocarcinoma in Asian patients and provides a comprehensive mutational landscape for both primary and metastatic tumours. This may thus form a basis for personalized medical care and shed light on the molecular pathogenesis of metastatic lung adenocarcinoma.

## Abstract (web summary)

Despite lung adenocarcinoma having a high global mortality, the genetic mutations present in Asian patients are uncharacterized. Here the authors use genomic and transcriptomic analysis to identify thirteen significantly affected genes, including *RHPN2*, *GLI3*, *MRC2*, *TP53* and *IQGAP3*.

## Graphical abstract

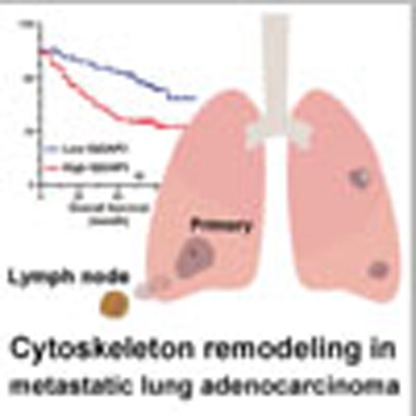

Lung cancer is the leading cause of cancerous deaths worldwide^1^,^2^ with two major types: non-small-cell lung cancer (NSCLC) and small cell lung cancer (SCLC), accounting for ∼85% and 15% of all diagnosed lung cancers, respectively^3^. Lung adenocarcinoma is the most common histological type of NSCLC, resulting in >500,000 deaths globally every year^4^. Despite advances in surgery, molecular subtyping and targeted therapy, prognosis of lung adenocarcinoma remains poor and the reasons for this could be due to: (1) Diagnosis was often made already at a late stage when localized malignant tumours spread to regional and distant tissues^3^; (2) Lack of known targetable driver genes in approximately half of the diagnosed patients^5^,^6^; (3) Complexity of inter- and intra-tumour heterogeneity^7^,^8^; and (4) Poor understanding on the mechanism of metastasis development, as well as lack of corresponding treatment.

Previous studies have characterized the genomic landscape of lung adenocarcinomas and identified many potential cancer driver genes^4^,^9^,^10^,^11^, of which targeting therapies have been developed for several activated oncogenes such as *EGFR*, *ERBB2* and *BRAF*^6^,^12^,^13^,^14^ and translocations or fusions involving *ALK*, *ROS1* and *RET*^15^,^16^,^17^. Most of these studies, however, mainly focused on tumour samples obtained from European or North American patients, and the majority of specimens were collected at early disease stages. Undoubtedly, it is important to have a comprehensive genomic analysis on lung adenocarcinomas from Eastern Asian population when considering its rapidly increasing incidence rate and potential genetic heterogeneity between different ethnic populations. Moreover, genetic characterization of advanced lung adenocarcinomas especially those harbouring corresponding metastases will not only expand the spectrum of potential cancer driver genes involved in lung carcinogenesis, but also improve our knowledge on metastasis formation and further guide diagnosis and therapies for metastatic lung adenocarcinomas. A recent study demonstrated high concordance of recurrent somatic alterations between primary tumours and matched metastases in NSCLCs^18^. This initial survey was limited though by focusing on targeted sequencing of 189 cancer-related genes.

Here we performed a comprehensive genetic analysis of 101 Chinese lung adenocarcinomas, as well as 35 corresponding lymph node metastases through multiplatform sequencing. Two hundred and thirty-four primary tumours were furthermore included into this study as an independent validation cohort. In an addition to a number of previously reported lung cancer driver genes, we have identified several novel, potentially oncogenic genes that are significantly mutated in our cohort. Integrative analyses through genomic data furthermore highlight pathways that may play a critical role in driving tumour metastasis. These results provide new insights on the pathogenesis of lung adenocarcinomas and also form a basis for further improvement of clinical management of our patients in the precision medicine era.

## Results

### Sample description and sequencing statistics

Paired tumours and normal adjacent tissues were obtained from 335 patients that provided written informed consent to carry out genomic studies in accordance with local Institutional Review Boards. All tumour specimens were reviewed by independent pathologists to determine the histological subtype, TNM stage and tumour cellularity (Supplementary Fig. 1). Detailed clinical features were summarized in Table 1 and Supplementary Data 1.

Whole-genome, transcriptome sequencing data were obtained from primary tissues and corresponding lymph node metastases of 24 Chinese lung adenocarcinoma patients. Genomes were sequenced to a mean depth of 49.6 × (range: 42.0–57.8 ×) for primary tumours and 51.2 × (44.4–66.3 ×) for metastatic specimens, while it was 31.9 × (range: 23.6–34.9 ×) for adjacent normal tissues. On average 93M clean reads (73–110M) were generated from whole-transcriptome sequencing. Whole-exome sequencing (WES) was performed on both primary and matched lymph node metastases from an additional 11 cases, and from primary tumour samples from a further group of 66 patients. RNA-seq analysis was furthermore performed in 32 cases from the latter group. Exome sequencing reached median fold coverage of 81.4 × (37.6–293.7 ×) and 37.3M reads (20.0–53.4M) were generated from RNA-seq (Supplementary Data 2 and 3). These 101 cases constituted the discovery cohort.

For verification, an independent hybrid-recapture and ultra-deep DNA sequencing were performed on a set of 51 selected genes with a mean fold coverage of 140 × (48.2–455.1 ×) on custom target regions in 98 cases of the discovery cohort described above and additional 234 primary lung adenocarcinomas (the validation cohort) (Supplementary Data 4). The cohort description with different data sets was summarized in Supplementary Data 5.

### Somatic DNA alterations and verification

Somatic point variations and small insertions/deletions (indels; <50 bp) were detected using MuTect^19^ and Platypus^20^, respectively. A mean of 9.7 somatic mutations per Mb (1.7–64.4) were identified from the first 101 primary lung adenocarcinomas, while the mean somatic mutation rate in 35 lymph node metastases was 6.4 mutations per Mb (0.8–60.0) (Supplementary Data 6 and 7). A significantly higher mutation rate was observed in smokers (mutations per Mb: mean 14.2, range 2.3–64.4) than in non-smokers (mutation per Mb: mean 7.0, range 1.7–29.2, *P*=0.0144, Wilcoxon rank sum test) for primary tumours, while the mutation rate in metastases was 9.2 (0.8–60.0) versus 4.7 (1.2–12.1) for smokers and non-smokers, respectively.

Identified mutations were verified through two procedures. First, mass spectrum was performed by using the MassArray platform of Sequenom to validate 79 randomly selected mutations, of which 76 (96.2%) were confirmed (Supplementary Data 8). Otherwise, ultra-deep target region sequencing on a set of 51 selected genes was used to validate the mutations identified in 98 primary tumours and 33 metastasis samples. In this analysis, a total of 967 single nucleotide polymorphisms (SNPs) and indels that passed pipeline filters were assayed, of which 960 were successfully verified, achieving a validation rate of 100% and 96% for single nucleotide variants (SNVs) and indel, respectively (Supplementary Data 9).

### Mutational signatures of lung adenocarcinomas

**Figure 1:**
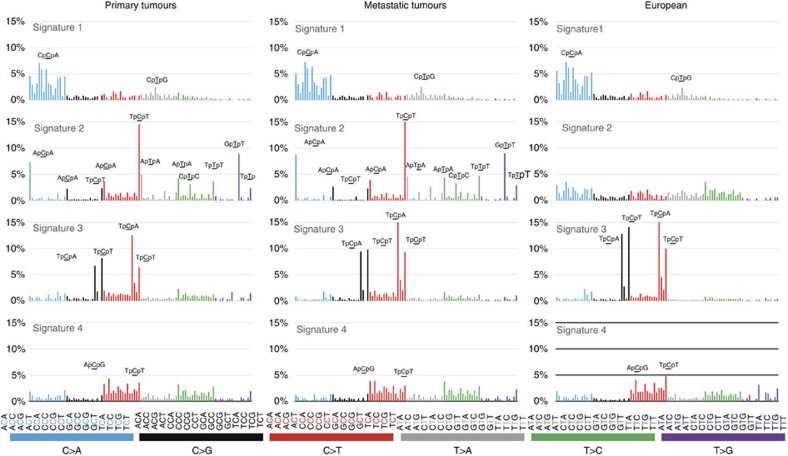
Mutational signatures of lung adenocarcinoma. Comparison of signatures between primary and metastatic lung adenocarcinomas in this study as well as lung adenocarcinomas derived from a previously published European cohort. Signatures were displayed according to the 96-substitution classification, with *x*-axes showed mutation types and *y*-axes showed trinucleotide frequency of each mutation type.

The somatic mutation detection in our discovery cohort reveals a consistently higher mutation rate in lung cancer than the other tumour types except malignant melanoma, which links to exposure to ultraviolet light^21^. Mutation spectrum analysis revealed that the most common somatic substitutions in our discovery cohort were C→A transversions and C→T transitions as previously reported for lung adenocarcinomas^4^ (Supplementary Fig. 2). Mutational signatures of 24 cases with whole-genome sequencing (WGS) data were subsequently characterized on both primary and metastatic tumours based on the 96 possible mutation types and were compared with the WGS data derived from previously reported European lung adenocarcinomas^4^. Four highly confident mutational signatures were extracted from each sample set and showed no apparent difference between primary and metastatic samples (signature 1, 2, 3, 4) (Fig. 1). When compared with the four signatures extracted from European lung adenocarcinomas, highest correlation (Pearson Correlation >0.99) was detected in signatures that were predominated by C>A mutations (Chinese signature 1 and European signature 1) and associated with cigarette smoking. The second highly correlated signature was Chinese signature 3 to European signature 3 (Pearson correlation >0.95), which attributed to over-activated members of the APOBEC family of cytidine deaminase. Indeed the expression levels of APOBEC1, APOBEC3B, APOBEC3C and APOBEC3F were found to be significantly higher in both primary and metastatic tumours as compared with normal tissues (*P*<0.01, Supplementary Fig. 3). Similar analysis was also applied to 10 previously reported stage I Chinese lung adenocarcinomas with WGS data^22^. Three high confident signatures from these tumours were identified, which show high correlation with signature 1, signature 3 and signature 4 that were extracted from 24 Chinese lung adenocarcinomas at late stage and 22 European lung adenocarcinomas. This observation indicated the significant association of these three signatures with lung adenocarcinomas, at both early and late stage of the disease, and they are shared within different ethnic populations. It is notable that signature 2, with transitions and transversions that predominantly occur at ApNpA and TpNpT trinucleotides (Fig. 1), is specific to our cohort that was not found in any of the 30 cancer types reported before^21^ or in early stage lung adenocarcinoma. Furthermore, the metastatic tumours seem to more often (46%, 11/24) carry dominant proportion of ‘signature 2' (with >20% of contribution) as compared with the primary tumours (25%, 6/24). The contribution of ‘signature 2' is highly correlated with the proportion of indel mutations, as tumours carried signatures with higher contribution of ‘signature 2' also harboured significantly higher proportion of indel mutations in all somatic mutations detected (Supplementary Fig. 4a). In contrast, the other signatures do not show such linear association with indel mutation. Since the mutational signature analysis was carried out only using base substitutions, the association of indel mutation of specific signature should not be attributed to incorporation of indel mutations to the analysis. We then verified our finding in the mutation data of 22 European lung adenocarcinomas. We found that the contribution of ‘signature 2' in these 22 European lung adenocarcinomas is only 4% on average, significantly lower than that in primary tumours (18%) and metastatic tumours (22%) in our sequenced cohort (*P*<0.001, Wilcoxin Signed Ranks Test). This explained that why this signature was not evidently identified from the previous analysis in the European sample set. However, we confirmed that in the European tumours, the contribution of ‘signature 2' is also highly correlated with the proportion of indel mutations (Supplementary Fig. 4b). No germline or somatic mutations in DNA repair genes including various mismatch repair genes, *BRCA1* and *BRCA2* could explain the occurrence of ‘signature 2'. Although this signature seems to be due to inclusion of large number of late stage of lung adenocarcinoma patients with metastasis in our study, WGS data in a larger cohort of patients will be required to confirm this association and to further understand the underlying mutagenesis mechanism.

Distinct combinations of mutational signatures were observed in each individual cancer, which indicated inter-tumour heterogeneity (Supplementary Fig. 5a). It is interesting that though we identified similar landscape of mutational signatures between primary and metastatic sample sets, about 33% (8/24) cases showed different contribution of signatures between the paired primary and metastatic tumours, which may be attributed to intra-tumour heterogeneity or mutational selection during metastases (Supplementary Fig. 5b). No significant correlation was observed between the mutation signature distribution and patient age, gender, smoking status or tumour purity (Supplementary Data 10), which may be partially due to the limited sample size.

### Significantly mutated genes

**Figure 2:**
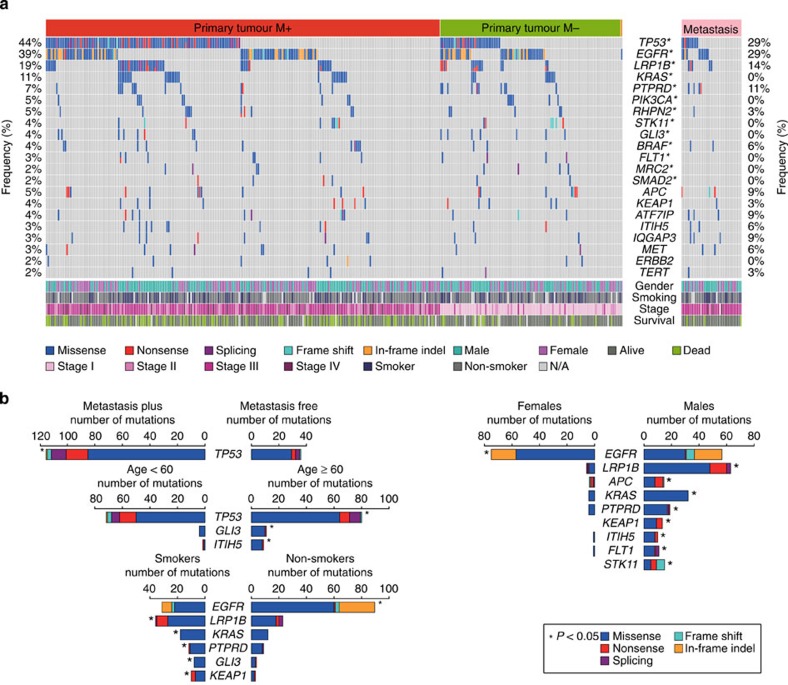
Somatic mutations and clinical association in lung adenocarcinomas. (**a**) Recurrently mutated genes and mutant frequencies in the full discovery and validation cohorts, comprising 335 primary tumours and 35 metastatic tumours. Primary tumours were classified into two groups: samples with metastases in adjacent lymph nodes or distant organs on diagnosis or surgery (PM+, *n*=229), and samples which were metastasis free at the time for diagnosis (PM−, *n*=105). Gender, smoking status and tumour stages were listed at the bottom according to the samples, as well as mutation types. Asterisks indicate genes predicted to be significantly mutated by MutSig algorithm (FDR<0.1). (**b**) Associations of specific mutated genes with metastasis status, gender, smoking status and age. Asterisks were marked at the sides of sample sets with significantly higher mutant frequencies (*P*<0.05, Fisher's exact test).

Given the high level of background mutation rate in lung cancer, oncogenic driver event analysis was carried out through a modified pipeline described previously^23^,^24^, which considered the mutation prevalence in the context of the background mutation rate and gene sequence length, as well as evaluation of functional impact. The MutSig^25^ algorithm was subsequently applied to identify significantly mutated genes and the mutant frequency was calculated by integrating the discovery and validation cohorts, for altogether 335 primary tumours and 35 metastasis specimens. These analyses revealed 13 statistically significant mutated genes (*q*<0.1, Fig. 2a, Supplementary Fig. 6, Supplementary Data 11). The most frequently mutated genes in primary tumours are *TP53* (44%), *EGFR* (39%), *LRP1B* (19%) and *KRAS* (11%), indicating the major contribution of these genes in lung carcinogenesis. The other frequently mutated genes include well-known tumour suppressor genes: *PTPRD* (7%), *STK11* (4%) and *SMAD2* (2%), and oncogenes *PIK3CA* (5%), *BRAF* (4%) and *FLT1* (3%). Consistent with the previous studies, *KRAS* mutations are mutually exclusive with those of *EGFR*, and are more commonly observed in smokers^4^,^10^,^26^. We also confirmed a higher mutation rate of *EGFR* than *KRAS* in our cohort, which is in contrast to that in the Caucasian populations. Notably, 54% (71/132) of the *EGFR* mutations are Leu858Arg, and another 29% (39/132) *EGFR* mutations are exon 19 deletions, which are sensitive targets of tyrosine kinase inhibitor therapies^12^. This confirms the importance of testing these specific mutations for Chinese lung adenocarcinoma patients.

Mutations in *RHPN2* (5%), *GLI3* (4%) and *MRC2* (2%) have not been reported previously as driver genes in lung adenocarcinomas but were recurrently observed in our cohort and are nominated to be significantly mutated. *RHPN2*, which encodes a member of the rhophilin family of Ras-homologous (Rho) GTPase-binding proteins, was first identified to interact with RhoA as a downstream effector molecule to regulate the actin cytoskeleton^27^, a process that is involved in cancer cell migration and invasion. A recent study further demonstrated that RHPN2 could drive mesenchymal transformation in malignant glioma via triggering RhoA activation^28^. Notably, a novel but recurrent mutation V73M was identified for *RHPN2* (Supplementary Fig. 6b), which affecting the Rho binding domain, implying that this site could be a potentially functional important hotspot mutation. *GLI3* encodes a zinc-finger transcription factor that modulates the sonic hedgehog (SHH) pathway, and one recent study on NSCLC demonstrated that overexpression of truncated GLI3 was significantly associated with lymph node metastasis and poor survival^29^. Another investigation also showed that high expression of GLI3, together with GLI1, is associated with tumour progression in advanced lung adenocarcinoma^30^. The exact mechanism of *GLI3* mutations in tumorigenesis and whether the potentially affected SHH signalling could be targeted have not been determined. MRC2 (also known as uPARAP, Endo180 or CD280), a member of mannose receptor family, is found to be involved in extracellular matrix remodelling by mediating collagen degradation^31^,^32^. Functional experiments and clinical surveys have demonstrated that high expression of *MRC2* can promote tumour growth and drive metastasis, results in significantly worse prognosis in several cancers^33^,^34^,^35^,^36^.

Additional recurrently mutated genes that did not reach a statistical significance by MutSig, but may functionally impact carcinogenesis, were also identified. These include *APC* (5%), *KEAP1* (4%), *ATF7IP* (4%), *ITIH5* (3%), *IQGAP3* (3%), *MET* (3%), *ERBB2* (2%) and *TERT* (2%) (Fig. 2a, Supplementary Fig. 6 and Supplementary Data 11), of which *KEAP1* was mutually exclusively mutated with *EGFR*. The mutation frequencies of all the mutated genes described above in metastatic specimens are also shown in Fig. 2a. It is interesting to note that though *KRAS* was frequently mutated in primary tumours, no mutations were called for this gene in the 35 sequenced metastases, including those 5 samples with paired, *KRAS*-mutated primary tumours. The five metastatic tumours with *KRAS* mutations ‘loss' were first confirmed as genetically identical with the corresponding primary tumours, by comparing both the pre-sequencing mass spectrometric fingerprint genotyping (Supplementary Data 12) and the genome-wide SNPs called from sequencing data. Visual review of the sequencing reads in these samples subsequently confirmed the sufficient coverage of all mutated sites for high quality mutation calling (Supplementary Data 13).Visual inspection furthermore confirmed that none of the normal tissues carried any reads of the mutated *KRAS* allele. At most two or three reads with mutation can be visualized, however, from metastatic specimens, thus suggesting a significantly lower frequency (0.01 on average; range: 0–0.02) in the metastases as compared with the corresponding primary tumours (0.30 on average; range: 0.18–0.46). Sanger sequencing further showed evident mutant genotype in primary tumours but no peak of altered signal was detected either in normal tissues or metastatic tumours.

The mutation patterns were further correlated with other clinical features such as gender, smoking status, age and tumour stage. The *EGFR* mutations were more frequently observed in females, while mutations in *LRP1B*, *KRAS*, *PTPRD*, *APC*, *STK11*, *KEAP1*, *ITIH5* and *FLT1* were significantly enriched in males (*P*<0.05, Fisher's exact test). While *EGFR* mutations were enriched in non-smokers, mutations of *LRP1B*, *KRAS*, *PTPRD*, *KEAP1* and *GLI3* were significantly enriched in smokers (*P*<0.05, Fisher's exact test). Notably, *TP53*, *GLI3* and *ITIH5* were significantly more mutated in patients aged >60 years (*P*<0.05, Fisher's exact test) but none of the recurrently mutated genes was associated with tumour stage (Fig. 2b).

Metastasis is one of the most critical issues for disease progression in lung adenocarcinoma, however, whether the mutation patterns are different between tumours with or without metastasis is yet to be studied. All the samples were thus classified into two groups, those who had metastases in adjacent lymph nodes or distant organs on diagnosis or surgery (PM+, *n*=229), and those who were metastasis free at diagnosis (PM−, *n*=105). This classification was not associated with gender (*P*=0.64), smoking status (*P*=0.79) or age (*P*=0.08). Fisher's exact test indicated that *TP53* was the only gene that was significantly (*P*<0.05) enriched in patients harbouring metastases (Fig. 2b), suggesting an important role of this tumour suppressor gene not only in primary tumorigenesis but also in driving the metastatic process.

Kaplan–Meier survival analysis was performed in all patients (*n*=335) and validation cohort (*n*=234), respectively, to explore the potential association between the recurrently mutated genes and individual outcome. Individuals harbouring somatic mutations in *TP53*, *LRP1B*, *STK11*, *KEAP1*, *BRAF*, *MET* and *MRC2* had significantly shorter survival time than individuals with wild-type genotypes (Supplementary Fig. 7), which suggested that alterations in these genes could be used as prognostic markers in clinical practice.

### Somatic CNVs and mRNA expression profiling

**Figure 3:**
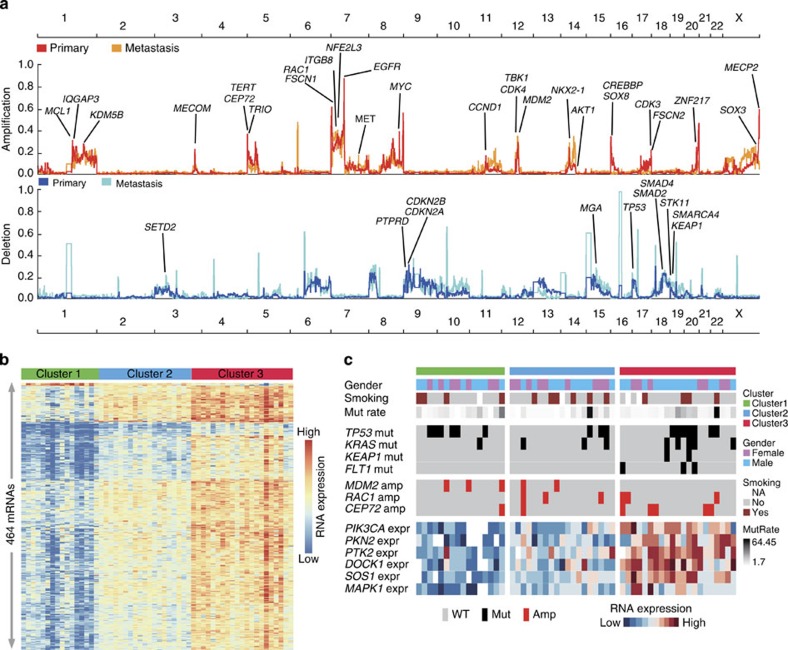
Genomic copy number alterations and mRNA expression profiling. (**a**) Landscape of genomic copy number alterations in Chinese lung adenocarcinomas. Amplifications and deletions across chromosome 1–22 and X were shown with *y*-axis presenting G-score altitude. CNV profiles in primary and metastatic tumours were shown with different colours. Putative cancer driver genes were marked in locations with peaks across the genome. (**b**) Cluster classification of 56 tumours indicated three clusters with different gene expression pattern, 464 representative genes are included. (**c**) Gene expression clusters integrated with genomic mutations. Tumours were ordered as three clusters shown in **b**. Alterations of selected genes were shown across clusters, revealing mutations (Mut.) of *KRAS*, *KEAP1*, *FLT1* as well as copy number amplification (Amp.) of *CEP72* were enriched in cluster 3. Cluster 3 was also characterized as having exceeded expression (Expr.) status in genes participating in PI3K–Akt pathway or cytoskeleton remodelling process.

Somatic copy number alterations (SCNAs) were profiled in the 101 primary tumours and 35 metastases from the discovery cohort. The results revealed frequent abnormalities of chromosomal arms involving gain of 1q, 3q, 5p, 7p/q, 8q, 14q, 16p, 17q and 20q, as well as loss of 3p, 4q, 6q, 8p, 9p, 12q, 13q, 15q, 17p and 18q (Supplementary Fig. 8), most of which were consistent with previous results reported in lung adenocarcinomas^9^. GSITIC^37^ was applied to identify statistically significant recurrent focal copy number variants (CNVs) (Fig. 3a, Supplementary Data 14). Significant somatic amplifications of *NKX2-1*, *TERT*, *EGFR*, *CCND1*, *MDM2*, *CDK4*, *MET*, *MYC* and *MECOM* were observed, as well as deletion of *TP53*, *PTPRD* and *CDKN2A*/*2B* that have been widely reported in lung adenocarcinoma. Significantly amplified regions that encompassed genes involved in cytoskeleton organization or focal adhesion were also identified, including *IQGAP3*, *TRIO*, *FSCN1/FSCN2* (fascin homologue 1/2, actin-bundling protein), *RAC1/RAC3* and *ITGB4/ITGB8*. Recurrent, novel focal deletion regions were furthermore detected affecting tumour suppressor genes *SMAD2* and *SMAD4*. Profiling of CNAs in metastatic tumours additionally identified amplification of *SOX3* and deleted regions affecting *STK11*, *KEAP1* and *MGA*. It is interesting that CNAs of several genes with function in histone or chromatin modification, such as *KDM5B*, *CREBBP*, *SETD2*, *SMARCA4* and *MECP2* were also found either in primary or metastatic tumours, suggesting a potential role of epigenetic regulation in lung adenocarcinoma. Profiling of mRNA expression status of 56 tumour/adjacent normal pairs confirmed correlated higher expression level in genes with copy number amplification than those with deleted CNAs (Supplementary Fig. 9).

Unsupervized clustering analysis of gene expression data from the 56 primary tumours identified three subgroups (cluster 1, cluster 2 and cluster 3) (Fig. 3b). Kaplan–Meier survival analysis indicated that cluster 3, which has higher frequencies of *KRAS*, *FLT1*, *KEAP1* mutations and amplified *CEP72*, demonstrated a significantly shorter survival time than patients in the other two clusters (Supplementary Fig. 10). We also found that several genes participate in the PI3K–Akt pathway or cytoskeleton remodelling process, such as *PIK3CA*, *PKN2*, *PTK2*, *DOCK1*, *SOS1* and *MAPK1* had elevated expression status in cluster 3 as compared with cluster 1 and cluster 2, implying the activation of these processes may direct to disease progression (Fig. 3c).

### Identification of structural alterations

Taking advantage of high depth WGS (50 × coverage on tumour samples and 30 × coverage for adjacent normal tissues) and corresponding transcriptome sequencing of 24 cases with paired primary and metastasis tumours sequenced, the structural alterations in lung adenocarcinoma were characterized. A total of 2,287 and 1,910 somatic structural aberrations were detected from primary and metastatic tumours, respectively (Supplementary Data 15 and Supplementary Fig. 11). Among the translocation events in primary tumours, 1,404 were inter-chromosomal (CTX), 201 were intra-chromosomal (ITX), 295 were inversions (INV) and 387 were deletions (DEL). The numbers of aberrant events in metastases were 1,467 CTXs, 127 ITXs, 107 INVs and 211 DELs. Well-known fusion genes *EML4-ALK* were identified in 2 out of these 24 cases in both primary and metastatic tumours with supportive evidence from both DNA and RNA data. Further screening through targeted sequencing of all 335 cases revealed 7 additional tumours harbouring this gene–gene fusion, giving an overall 3% alteration rate. Concurrent and mutual exclusion analysis indicated that *EML4-ALK* fusion was mutually exclusive with *EGFR* mutations (Supplementary Fig. 12), consistent with a recent large-scale clinical screening of NSCLC for *EGFR*, *KRAS* mutations and *ALK* rearrangements^38^. Previously reported rearrangements of *ROS1* and *RET* genes in NSCLC patients were not observed in our patient cohorts.

We further identified additional 13 putative gene–gene fusion events in these 24 metastatic cases using a combination of the whole-genome and transcriptome sequencing data (Supplementary Data 16, Supplementary Fig. 13). It is notable that among these fusions, several genes that function in cytoskeleton regulation were involved, including *IQGAP3*, *EPB41*, *CDC42*, *PARD6G*, *PTK2B* and *KALRN*, suggesting that these alterations may promote metastasis development. Other fused genes with putative role in tumorigenesis or metastasis formation include *CHEK2*, *FGFR2*, *MIIP*, *CPB1*, *EIF4A2*, *MECOM* and *USP32*.

### Analysis of the *IQGAP3* locus

Integrated analysis of somatic mutations, CNAs, structural aberrations and gene–gene fusions using multiplatform studies highlighted *IQGAP3* was altered in a notable number of cases. This gene, a member of the IQGAP family that has both IQ motifs and Ras GTPase-activating protein-related domain (GRD)^39^,^40^, was altered in 10% of lung adenocarcinomas (discovery cohort, *n*=101), by somatic mutations (three cases), copy number amplification (seven cases) and translocation (one case). Targeted regional sequencing of the validation cohort (*n*=234) further identified eight additional cases harbouring somatic mutations on *IQGAP3*.

**Figure 4:**
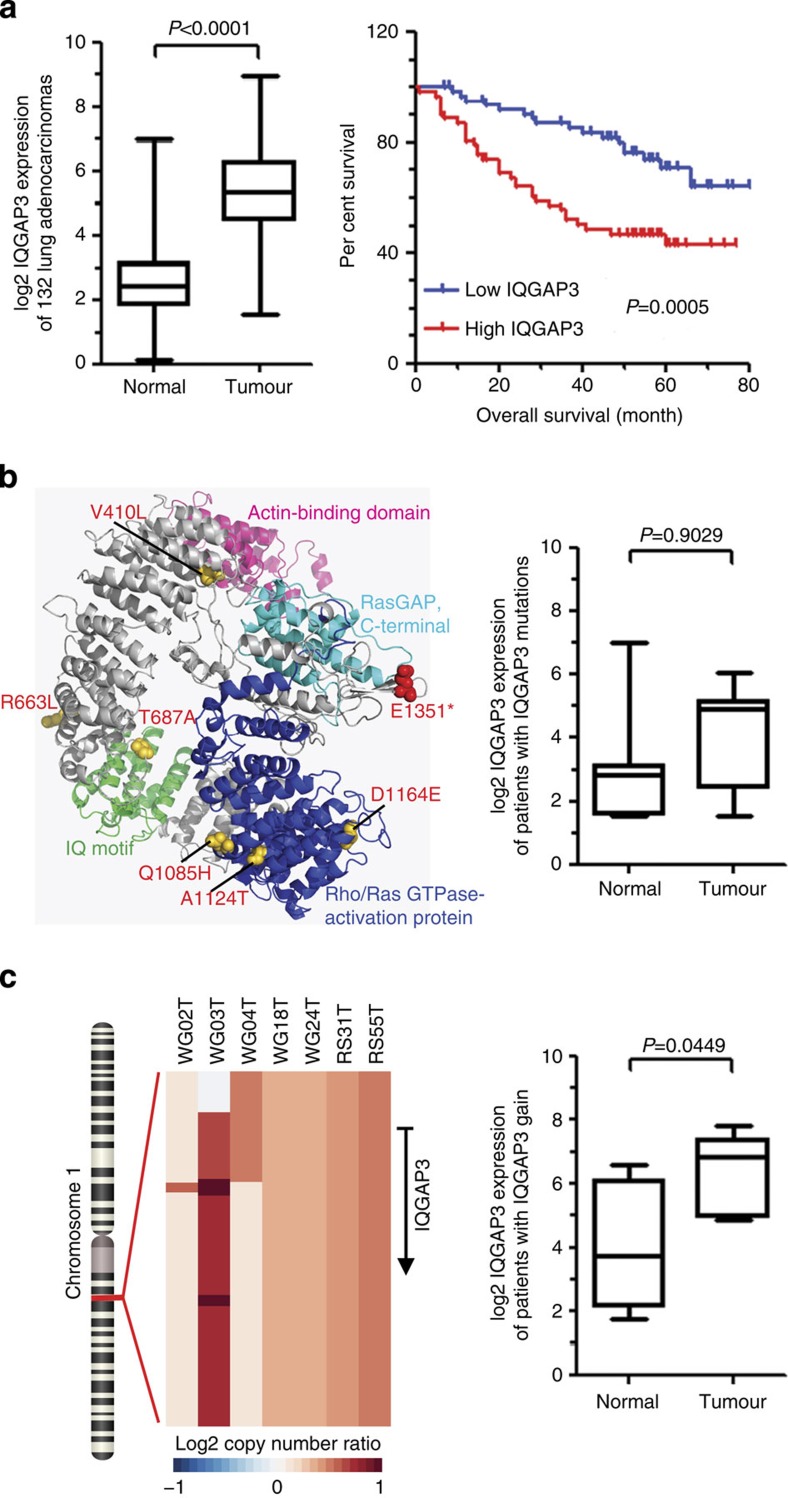
Integrated characterizations of *IQGAP3* alterations. (**a**) Quantitative RT–PCR analysis of 132 lung adenocarcinoma patients showed significantly higher expression of IQGAP3 mRNA in tumours than in adjacent normal tissues in lung adenocarcinoma patients. Kaplan–Meier survival curves showed that patients with high IQGAP3 expression had shorter overall survival. (**b**) Three-dimensional model of IQGAP3 protein showing the somatic mutations affecting functional domains. qPCR analysis in patients harbouring these mutations showed no significant difference of IQGAP3 expression between tumour and normal tissues. (**c**) Tumours harbouring genomic amplifications of *IQGAP3* in chromosome 1. QPCR analysis indicated higher expression of IQGAP3 in corresponding tumours with copy gain of *IQGAP3* than in adjacent normal tissues.

Analysis of mRNA expression of 56 RNA sequenced primary tumours and 24 RNA sequenced metastases revealed that *IQGAP3* was expressed at a significantly higher level in tumour specimens than adjacent normal tissues (Supplementary Fig. 14). The *IQGAP3* expression in 132 primary lung adenocarcinomas (61 from discovery cohort and 71 from validation cohort) with available RNA within the 335 sequenced cases was subsequently measured by quantitative reverse transcription–PCR (RT–PCR; Supplementary Data 17), and confirmed the higher expression level of *IQGAP3* in these tumours (Fig. 4a). An elevated level of IQGAP3 protein was also confirmed in a subset of primary tumours by immunohistochemical staining (*n*=42, *P*<0.001, Wilcoxin Signed Ranks Test, Supplementary Fig. 15). Kaplan–Meier analysis indicated that patients with a higher level of *IQGAP3* mRNA had significantly shorter overall survival and disease-free survival (Fig. 4a, Supplementary Fig. 16). Furthermore, Multivariate Cox proportional hazard analysis showed that a higher level of IQGAP3 was an independent risk factor for poor prognosis of the lung adenocarcinoma patients (Supplementary Data 18 and 19, hazard ratio=2.28, 95% confidence interval=1.25–4.14, *P*=0.007).

The association of *IQGAP3* genetic alterations and mRNA expression was subsequently investigated. No significant difference was observed in *IQGAP3* expression between tumours and normal tissues in the eight cases carrying somatic mutations, including four mutated sites located in the Ras GTPase-Activating Protein (RasGAP) domain (Q1085H, A1124T, D1164E and L1292 splicing site) and one truncated mutation that resulted in the loss of the RasGAP C terminus domain (Fig. 4b). Furthermore, patients harbouring *IQGAP3* SNVs showed better survival status than the other cases though did not reach a statistical significance (Supplementary Fig. 7). By contrast, this gene was overexpressed in tumours affected by copy number amplifications (Fig. 4c). An inter-chromosomal rearrangement occurred between chromosome 17 and chromosome 1 was identified in patient WG04, in both primary and metastasis tumours, which results in a novel gene–gene fusion involving *TMC6* and *IQGAP3*, with reversed 5′ and 3′ orientation (Supplementary Fig. 17a). The fused product consists of exons coding most of the RasGAP domain and the intact RasGAP C terminus domain of *IQGAP3*, while retained exon 1 and exon 2 of *TMC6*. RNA-seq data revealed markedly higher, outlier expression of *IQGAP3* in primary and metastasis tumour of patient WG04, and investigation of reads coverage indicated that highly covered regions were enriched in exons containing RasGAP and RasGAP C terminus domains (Supplementary Fig.17b,c).

### Altered key pathways and therapeutic targeting

**Figure 5:**
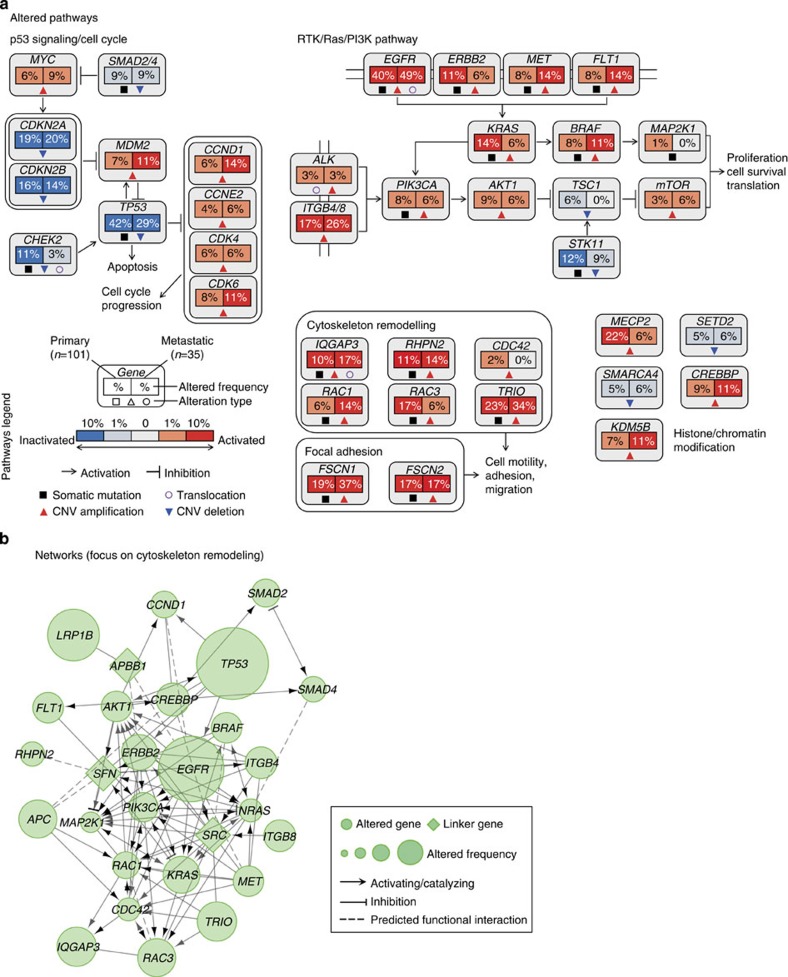
Altered pathways and network in lung adenocarcinomas. (**a**) Somatic mutations, copy umber alterations and genomic rearrangements affecting p53 signalling/cell cycle process, RTK/Ras/PI3K pathway, cytoskeleton remodelling regulation and histone/chromatin modification. Percentage presented alteration frequencies in 101 primary tumours and 35 metastatic tumours, respectively. (**b**) Network connection of genes involved in cytoskeleton remodelling regulation. Gene–gene interactions are inferred by CytoScape program.

**Figure 6:**
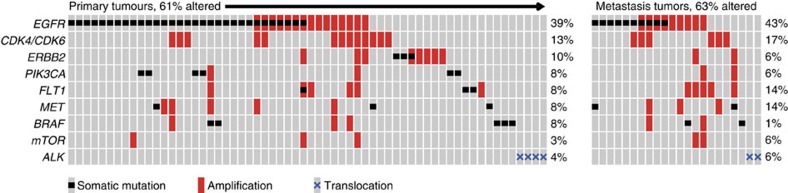
Therapeutic targeting in lung adenocarcinomas. Somatic mutations, copy number alterations and translocations affecting genes that are regarded as targets of specific antibodies or kinase inhibitors. Somatic mutations only consist of recurrent mutations in this cohort or mutations previously reported in the COSMIC database. Primary tumours and metastatic tumours with at least one alteration are shown.

Integrative analysis of altered key pathways affected by somatic mutations, CNAs as well as frequent rearrangements was performed to construct a comprehensive view of aberrant characteristics for lung adenocarcinoma (Fig. 5a). The most frequently altered pathways in primary tumours were p53 signalling and cell cycle process, affecting 87% of primary tumours and 89% of metastatic tumours. A majority of the observed mutations were somatic mutations in *TP53*, amplifications of *MDM2*, *MYC*, *CCND1*, *CCNE2*, *CDK4* and *CDK6*, as well as loss-of-function variants including deletions of *CDKN2A*, *CDKN2B*, mutations and deletions affecting *SMAD2* and *SMAD4*, of which *TP53* mutations were mutually exclusive with *MDM2* amplification (Supplementary Fig. 12). *CHEK2*, a serine/threonine-protein kinase involved in regulating cell cycle arrest, was altered either by somatic mutation, deletion or rearrangement. The RTK/Ras/PI3K pathway was also frequently altered in lung adenocarcinoma, with 88% of primary tumours and 80% of metastasis tumours in this study harbouring aberrations in genes involved in this pathway. Included were events affecting several therapeutic targets that were responsive to antibodies or kinase inhibitors, such as alterations of *EGFR*, *PIK3CA*, *ALK*, *ERBB2*, *MET*, *AKT1* and *mTOR* (Fig. 6).

Specifically, an additional pathway that involves actin cytoskeleton remodelling and focal adhesion was frequently altered in lung adenocarcinoma, affecting 50% of primary tumours. In addition to *IQGAP3*, five other genes with important roles in cytoskeleton remodelling exhibited somatic mutations or copy number amplifications, namely, *RHPN2*, *RAC1*, *RAC3*, *TRIO* and *CDC42*. *FSCN1* and *FSCN2* were found to be frequently amplified in our studied cohort. These two genes belong to the Fascin protein family, which organizes F-actin into parallel bundles and plays an important role in cell migration, motility, adhesion and cellular interactions. Several studies have demonstrated a modulating function of Fascin proteins, especially FSCN1 in tumour metastasis and considered the overexpression of this gene as a prognostic marker for aggressiveness in carcinomas^41^,^42^,^43^. Network analysis further extended the interactive association between genes and pathways (Fig. 5b) that promote regulation of cytoskeleton remodelling. It is notable that this process was more frequently in metastasis samples (77%) than primary tumours (50%), implying the potential crucial contributions of these genes in driving metastasis.

## Discussion

Although lung adenocarcinoma is one of the most well-studied cancers worldwide, the accuracy of diagnostic and therapeutic effectiveness are far from satisfactory, especially for patients harbouring metastases. In the present study, we carried out a comprehensive genetic characterization of 335 lung adenocarcinomas by integrating WGS, WES and RNA-seq. Sixty-eight per cent of studied cases were metastasis positive, providing an opportunity for the investigation of potential mechanisms and identifying biomarkers for driving metastasis.

Our study first delineated the similarity and distinctive mutational features of lung adenocarcinomas between Chinese and Caucasians. We discovered signatures associated with smoking and activation of APOBEC molecules that were shared in tumours from both ethnic populations and also detected signature specific to our cohort, which consists mainly late stage lung adenocarcinomas and is associated with the proportion of indel mutations. *EGFR* was mutated at a much higher frequency in our cohort than in Caucasians, with a dominance of the Leu858Arg change. By contrast, *KRAS*, the second most commonly mutated gene in Caucasians, was only found in 11% of Chinese patients. Notably, we observed significantly lower frequency or even loss of *KRAS* mutation in the metastatic tumours as compared with their paired primary tumours. The discordance of *KRAS* mutations in primary tumours and corresponding metastases has been noted previously in a few cases of lung adenocarcinomas^44^,^45^. Independent studies demonstrated that oncogenic *KRAS* itself initiates the growth of non-invasive tumours in mouse models of lung cancer^46^,^47^, implying that *KRAS* mutations may induce tumorigenesis, but might not be responsible for tumour metastasis, at least in some cases. Alternative interpretation of such discordant observation could attribute to tumoral heterogeneity, thus analysis of more biopsies from both primary tumour and metastatic lymph nodes from the same individuals may give a more complete picture^48^. Our results suggest that alterations in major cancer drivers may change during tumour evolution and the development of metastasis, which should be important in clinical application, for instance, it may not be sufficient to determine therapeutic strategy for metastatic tumours base solely on the mutation spectrum in primary tumours.

We identified three genes, *RHPN2*, *GLI3* and *MRC2* to be significantly mutated in our studied cohort. These genes are altered in lower frequency, therefore are not determined as driver genes in lung adenocarcinoma previously. According to the saturation analysis by Lawrence *et al*.^49^, the discovery of candidate cancer genes is significantly associated with the sample set used and taking lung adenocarcinoma as an example ∼2,000 cases are required to achieve a comprehensive catalogue of cancer genes with sufficient statistic power when the genes are mutated in >3% of patients. Our discovery of novel significantly mutated genes in lung adenocarcinoma confirms the necessity of continuous sequencing and analysis effort on more tumour-normal pairs of cancer patients. Although our study presented the potential association of these mutations to lung adenocarcinoma through bioinformatic exploratory analysis, functional study is essential to demonstrate the exact mechanism of these mutated genes in lung adenocarcinoma carcinogenesis in the future.

We also showed a significant enrichment of *TP53* mutations in patients with metastases on diagnosis or surgery. *TP53* was in fact the most frequently mutated gene in many types of cancers. Emerging evidence indicates that mutant TP53 not only loses its tumour-suppressing activity, but also can gain functions that contribute to tumour progression^50^,^51^,^52^,^53^. Many mutations of *TP53* have been implicated in invasion, migration and metastasis in different tumour types (summarized in ref. 54).

Integrative pathway investigation revealed that the most commonly altered processes are related to p53 signalling/cell cycle progression and the RTK/Ras/PI3K (mTOR) pathways in lung adenocarcinoma. Within this group of molecules are several targets for kinase inhibitors that are successfully used for treatment of patients with sensitive mutations. Furthermore, the actin cytoskeleton remodelling process, along with genes involved in focal adhesion was observed to be frequently altered in lung adenocarcinoma. Among the mutated genes involved in cytoskeleton remodelling, *IQGAP3* was highlighted as numerous different alterations were identified including a novel fusion event. Clinical association analysis indicated that a high level of IQGAP3 expression is a putative biomarker in disease progression and poor survival status, implicating *IQGAP3* as a potentially targetable gene for novel therapeutic strategies. Overall our study provided the first comprehensive description of mutational landscape of lung adenocarcinoma in Chinese patients, defined potential mechanisms for cancer metastasis and provided a basis for effective personalized therapies for this lethal cancer. Whether the discovered novel genomic changes are specific for Asian population and/or represent the disease heterogeneity, remains to be further explored in larger sets of tumour samples.

## Methods

### Patients and clinical samples description

The primary tumour specimens, adjacent normal tissues and lymph node metastases (if available) were obtained from 335 patients with lung adenocarcinoma who underwent surgical resection in Department of thoracic surgery, the First Affiliated Hospital of Guangzhou Medical University. None of the patients were subjected to chemotherapy or radiotherapy before surgery. Tumours and adjacent normal tissues were frozen in liquid nitrogen and stored at −80 °C for further research. The tumour specimens were reviewed by two pathologists independently to determine the histological subtype, TNM stage and tumour cellularity. The primary tumour tissues containing at least 50% of tumour cells and the lymph node metastases with at least 30% of tumour cells were included in this study. Detailed clinical characteristics were summarized in Table 1 and Supplementary Data 1. The routine follow-up was conducted every 3 months and the median follow-up was 36 months. About 227 patients (68%) were alive at the time of last follow-up. This study was approved by the Institutional Review Board of the First Affiliated Hospital, Guangzhou Medical University, Guangzhou, China. All patients who participated in this study provided written informed consent.

### Genomic DNA and total RNA preparation

The genomic DNA from tissues was prepared using the QIAamp DNA Mini Kit (Qiagen) following the manufacturer's instructions. The total RNA was prepared using TRIzol Reagents (Life Technologies). The quantity and quality of DNA/RNA were determined by agarose gel electrophoresis or Agilent 2100 analyzer. Mass spectrometric fingerprint genotyping of 21 common SNPs was performed for all the samples before sequencing, to verify that paired tumour (primary and metastatic) and normal tissues were derived from the same patient.

### Whole-genome and WES

Prior to the library construction, 2–3 μg of genomic DNA from each sample was fragmented using a Covarias sonication system to mean sizes of ∼500 bp. After fragmentation, libraries were constructed according to the Illumina Paired-End protocol. Briefly, the purified, randomly fragmented DNA was treated with a mix of T4 DNA polymerase, Klenow fragments, T4 polynucleotide kinase and a nucleotide triphosphate mix to repair the ends by blunting and phosphorylation. The blunted DNA fragments were subsequently 3′-adenylated using the Klenow fragment (3′–5′exo) and ligated by T4 DNA ligase to BGI-designed PE Index Adaptors that had been synthesized with 5′-methylcytosine in place of cytosine. After each step, the DNA was purified using the QIAquick PCR Purification Kit (Qiagen). For exome sequencing, 3 μg genomic DNA from each sample was fragmented and ligated PE Index Adaptors then captured with SureSelect Human All Exon 50 Mb Kit as described in the protocol for the SureSelect Target Enrichment System for an Illumina Paired-End Sequencing Library. All the constructed libraries were sequenced on HiSeq 2000 platform using 2 × 100-bp paired-end reads.

After removing sequencing reads containing adaptor sequence and low-quality reads, which have too many Ns (>10%) and low-quality base (>50% bases with quality <5), the high quality paired-end reads were gapped aligned to the UCSC human reference genome (hg19) using BWA^55^. After the processes of fixing mate information, adding read group information, Picard (v1.54) (http://picard.sourceforge.net/) was used to mark duplicate reads caused by PCR. Then local realignment of the BWA aligned reads was carried out by using the Genome Analysis Toolkit (GATK)^56^.

After fundamental analysis, the potential somatic substitutions were identified by MutTect^19^ with default parameter based on paired alignment files (primary and normal, metastasis and normal).The raw lists of somatic indels were detected by Platypus^20^ based on default parameter. After this process, germline variants could be effectively removed.

The Somatic CNVs of sample pairs with WGS were identified by modified-method based on Segseq algorithm and copy number gain or loss status using thresholds of ≥2.5 copies for gain and ≤1.5 copies for loss. The somatic CNVs of sample pairs with WES were identified by the CONTRA^57^ algorithm. In the CONTRA algorithm, base-level log-ratios between the case and control were taken into consideration to eliminated GC bias and imbalanced library size effect. Target regions of <10bp with depth of coverage <10 were discarded. For the remaining regions, the mean log-ratio was calculated and the significant *P* value was then assigned. Then the circular binary segmentation was applied to the log-ratios of regions with default settings. Segmentation results were then used for the subsequent analysis. GISTIC^37^ algorithm was used to infer recurrently amplified or deleted genomic regions, using copy numbers in 100-kb windows. G scores represented the frequency and amplitude of amplifications or deletions of each genomic region.

Somatic SVs were identified by a modified pipeline developed based on CREST^58^ algorithm. All the detected variants were subsequently annotated by ANNOVAR^59^.

### Validation of somatic mutations

Identified mutations were validated either by mass spectrum or target region captured resequencing. For mass spectrum validation, specific primers were designed for PCR amplification and base extension that covered the mutation sites. Genotyping assay and base calling of successfully amplified products were performed on the MassArray platform of Sequenom. Ultra-deep target region sequencing on a set of 51 selected genes was used to evaluate the validation status of identified SNPs and indels. One microgram of genomic DNA from each sample for validation was used for hybrid capture and library construction. Libraries were then sequenced on HiSeq 2500 platform using 2 × 100 bp paired-end reads sequencing.

### Mutational signature analysis

Mutational signature analysis was performed as follows: briefly, the computational framework for mutational signatures deciphering was downloaded from http://www.mathworks.com/matlabcentral/fileexchange/38724. Mutational signatures were extracted from the WGS data of 24 primary tumours and corresponding metastasis tumours as follow: (1) Somatic base substitutions of each data set were classified into 96 possible mutated trinucleotides, as 6 types of substitution (C:G>A:T, C:G>G:C, C:G>T:A, T:A>A:T, T:A>C:G and T:A>G:C) × 4 types of 5′ base (A, C, G, T) × 4 types of 3′ base (A, C, G, T), to generate mutational catalogue. Then the prevalence of somatic mutations was calculated for each type of substitution. (2) Signatures of 15 mutational processes from the mutational catalogue were deciphered by the mutational signature framework. The number of signature extracted (*N*) is determined by estimating the signature reproducibility and reconstruction error rate and as described by Alexandrov *et al*.^60^, *N* here is the number where the lowest reconstruction error is achieved without decreasing the reproducibility. (3) The minimal set of mutational signatures was then determined to optimally explain the proportion of each mutation type found in the catalogue, based on reproducibility of their signatures and low error for reconstructing the original catalogue. Somatic mutations of WGS from 22 European lung adenocarcinomas were downloaded from ftp://ftp.sanger.ac.uk/pub/cancer/AlexandrovEtAl. Pearson correlation analysis was performed to compare signature patterns between primary and metastasis tumours in this study and those of European lung adenocarcinomas. Contributions of distinct signatures in individual tumour were also profiled and Pearson correlation analysis was performed to compare the signature contributions between paired primary and metastasis tumours^21^,^60^.

### Identification of potential significantly mutated genes

Considering the high background mutation rate in lung adenocarcinoma, analysis of significance of mutated genes was performed through two statistical procedures. First, a cancer driver gene prediction model^23^ was used to calculate the significance of detected mutations for each mutated gene. The mutation prevalence and functional impact was evaluated by this approach, and a background distribution of mutation score was assigned to each gene then. Taking together the background distribution of mutation score and test statistics of observed mutation scores across samples, a *P* value was computed for each gene and adjusted using the Benjamini–Hochberg method to further determine the false discovery rate (FDR). Only genes with FDR (*q*-value) <0.1 were selected for following analysis. A subsequent prediction of significantly mutated genes was performed using the MutSig^25^ algorithm and genes with FDR *q*-value <0.1 were regarded as significant.

### Transcriptome sequencing

About 20 μg total RNA from each sample was treated with RNase-free DNase I for 30 min at 37 °C (New England BioLabs) to remove residual DNA. Beads with oligo (dT) were used to isolate poly (A) mRNA. First-strand complementary DNA (cDNA) was synthesized using random hexamer-primer and reverse transcriptase (Invitrogen). The second-strand cDNA was synthesized using RNase H (Invitrogen) and DNA polymerase I (New England BioLabs). Then the cDNA libraries were prepared following the manufacturer's instructions and were sequenced on the HiSeq 2000 platform.

The raw reads filtering process include three criteria: (1) Remove reads with sequence adaptors; (2) Remove reads in which unknown bases are >10%; (3) Remove low-quality reads, which have >50% in one read. All subsequent analyses were based on clean reads.

Mapping clean reads to the human genome and transcriptome, the reference sequences used were genome and transcriptome sequences downloaded from the UCSC website (version hg19). The Clean data were respectively aligned to the reference genome and transcriptome by SOAP2. Less than five mismatches were allowed for each read in the alignment.

The gene expression level is calculated by using RPKM method (reads per kb transcriptome per million mapped reads), and the formula is shown as follows:


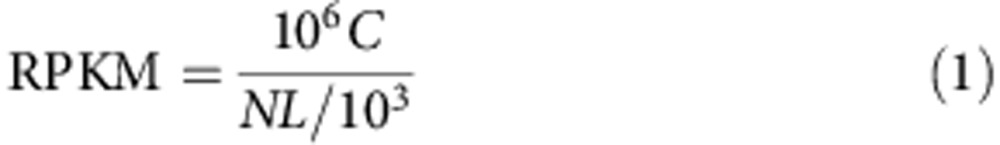


In which *C* is the number of reads that are uniquely mapped to the given gene, *N* stands for the total number of reads that are uniquely aligned to all genes and *L* is the total length of exons from the given gene.

### Identification and validation of fusion genes

Soapfuse^61^ was used to identify fusion genes between paired tumour and normal samples from transcriptome sequencing using the default parameters. Reads were mapped against the human reference genome sequence (hg19) and annotated transcript. SOAPfuse then detected two kinds of reads to identify potential fusion events: span-reads that were defined as paired-end reads mapping to two distinct genes and junction reads that covered the exact junction sites, after filtering duplicated reads a candidate fusion event was determined if more than two reads support the paired-end connection and junction site. WGS data of samples harbouring fusion genes were also analysed to confirm fusion events that were generated by genomic rearrangements with breakpoints in intron or exon regions. Confirmed fusion events were validated at both RNA and DNA level by PCR and Sanger sequencing.

### Concurrence and mutual exclusion analysis

Concurrence and mutual exclusion analysis was performed on significantly mutated genes, significant copy number variations and translocations or fusion events involving *ALK*, as well as clinical features such as smoking, age and metastasis status. Significance was calculated by Fisher's exact test.

### Pathway and network analysis

Pathway enrichment analysis was carried out by using WebGestalt^62^ to investigate the distribution of genes affected by somatic mutations, CNAs and genomic rearrangements within the KEGG database. To inquire the potential interaction network of altered genes, Cytoscape (V3.1.1) software^63^ (http://www.cytoscape.org/) was used to identify pathways and network patterns related to cancer through the reactome functional interaction plugin^64^.

### Real-time RT–PCR assay

Total RNAs of lung cancer cells and lung adenocarcinoma specimens were extracted using Trizol reagent (Invitrogen). The quality and quantity of RNA were determined by agarose gel electrophoresis and photospectrometry. One microgram of RNA was used for cDNA synthesis using the RT System (Promega) according to the manufacturer's instructions. Real-time PCR was performed using GoTaq qPCR Master Mix (Promega). Thermal cycling conditions were as follow: 1 step for 10 min at 95 °C followed by 40 cycles at 95 °C for 15 s and at 60 °C for 1 min. The quality of the PCR products was monitored using post-PCR melting curve analysis. Relative mRNA levels were calculated with ΔΔCt methods using β-actin for normalization.

### Immunohistochemistry

Immunohistochemistry analysis of IQGAP3 in the primary tumours of lung adenocarcinoma was carried out using a standard avidin–biotin–peroxidase technique. In brief, the formalin-fixed, paraffin-embedded tissue sections were deparaffinized and rehydrated through xylene, graded ethanol and water.

The endogenous peroxidase activity was blocked by incubating the sections in 3% hydrogen peroxide for 15 min, and heat-induced epitope retrieval was performed by incubating the sections in boiling sodium citrate buffer (10 mM sodium citrate, 0.05%Tween-20, pH 6.0) for 20 min in microwave. Then the sections were blocked with 10% normal goat serum in PBS for 10 min at room temperature, and incubated with primary antibody against IQGAP3 (1:80, ab151437, Abcam, USA) diluted in 1% BSA (Zhongshanjinqiao, Beijing, China) overnight at 4 °C.The Maixin Detection System Peroxidase/DAB, Rabbit/Mouse kit was applied to determine immunoreactivity according to the manufacturer's instructions, with 1:50 dilutions of DAB Chromogen to HRP Substrate Buffer. Sections were then washed, and counterstained for 30 s in filtered Harris Haematoxylin (Zhongshanjinqiao). After washing with PBS and dehydration with graded ethanol followed by xylene, the sections were mounted in neutral balsam (Zhongshanjinqiao).The slides were viewed and images were captured using Nikon Eclipse 80i microscope with NIS-Elements F software. Analysis of cytoplasm staining was performed by two pathologists independently through measuring the percentage of positively stained tumour cells. A consensus was reached for intensity by taking the mean if scores were within 20% of each other; otherwise, images were reviewed and a consensus reached.

### Statistical analysis for clinicopathological data

The correlation of IQGAP3 expression with clinicopathological characteristics was determined by Pearson's *χ*^2^-test. The differences of IQGAP3 mRNA and protein between tumours and normal tissues were tested by student's *t*-test and Wilcoxin signed rank test, respectively. Overall survival and disease-free survival were estimated using Kaplan–Meier method with log rank test. The prognostic significance of IQGAP3 was evaluated by Cox regression model with forward likelihood ratio (LR) method. All statistical analyses were performed using GraphPad Prism 5 and SPSS16.0.

## Additional information

**How to cite this article:** Wu, K. *et al*. Frequent alterations in cytoskeleton remodelling genes in primary and metastatic lung adenocarcinomas. *Nat. Commun.* 6:10131 doi: 10.1038/ncomms10131 (2015).

**Accession code:**

All the sequencing data of 335 lung adenocarcinomas have been deposited at the European Genome-phenome Archive (EGA, http://www.ebi.ac.uk/ega/), which is hosted by the EBI, under the accession code EGAS00001000982.

## Supplementary Material

Supplementary Information Supplementary Figures 1-17

Supplementary Data 1 Clinical Characteristics of each patient

Supplementary Data 2 Summary statistics of whole genome or whole exome sequencing data from 101 lung adenocarcinomas

Supplementary Data 3 Summary statistics of transcriptome or RNA-seq data from 56 lung adenocarcinomas

Supplementary Data 4 Summary statistics of target region sequencing data on 51 genes from 335 lung adenocarcinomas

Supplementary Data 5 Summary of cohorts and samples with different data sets

Supplementary Data 6 Statistics of somatic mutations and tumor cellularity of 101 lung adenocarcinomas

Supplementary Data 7 Somatic mutations identified in 101 lung adenocarcinomas

Supplementary Data 8 Verification of somatic mutations by mass spectrum

Supplementary Data 9 Verification of SNPs and Indels by target region sequencing on 51 genes across 131 tumors

Supplementary Data 10 Tumour Purity and Ploidy estimation of 24 pairs of primary lung adenocarcinomas and corresponding metastases with WGS

Supplementary Data 11 Somatic mutations identified in 335 lung adenocarcinomas

Supplementary Data 12 Mass spectrometric fingerprint genotyping of five primary tumors with KRAS mutations and corresponding lymph node metastases

Supplementary Data 13 Reads coverage of KRAS mutations in normal, primary and metastasis tissues

Supplementary Data 14 Somatic copy number alterations identified in 101 lung adenocarcinomas

Supplementary Data 15 Genomic arrangements identified through whole genome sequencing from 24 pairs of primary lung adenocarcinomas and corresponding metastases

Supplementary Data 16 Gene-gene fusion events supported by both whole genome and transcriptome sequencing data

Supplementary Data 17 Primers for quantitating IQGAP3 expression through quantitative RT-PCR

Supplementary Data 18 Multivariate Cox regression analysis of IQGAP3 expression and overall survival of lung adenocarcinoma patients

Supplementary Data 19 Correlation of IQGAP3 mRNA expression and clinicopathological characteristics of the patients with lung adenocarcinoma

## Figures and Tables

**Table 1:** Clinical feature summary of 335 sequenced lung adenocarcinomas.

	Discovery cohort	Validation cohort	Total	*P* value
Age at surgery, years				0.4918†
Median	59.2	58.6	58.8	
Range	25.2–81.6	32.4–84.9	25.2–84.9	
Gender				0.1303‡
Male	62 (61.4%)	121 (51.7%)	183 (54.6%)	
Female	39 (38.6%)	113 (48.3%)	152 (45.4%)	
Smoking status				0.3373‡
Smoker	37 (36.6%)	68 (29.1%)	105 (31.3%)	
Non-smoker	58 (57.4%)	141 (60.3%)	199 (59.4%)	
NA	6 (5.9%)	25 (10.7%)	31 (9.3%)	
Follow-up, months				0.1042†
Median	22	37	36	
Range	4–80	1–77	1–80	
Tumour stage				0.1447‡
I	19 (18.8%)	63 (26.9%)	82 (24.5%)	
II	18 (17.8%)	51 (21.8%)	69 (20.6%)	
III	56 (55.4%)	98 (41.9%)	154 (46%)	
IV	8 (7.9%)	21 (9%)	29 (8.7%)	
NA	0 (0%)	1 (0.4%)	1 (0.3%)	
Metastasis				0.1087‡
Negative	25 (24.8%)	80 (34.2%)	105 (31.3%)	
Positive	76 (75.2%)	153 (65.4%)	229 (68.4%)	
NA	0 (0%)	1 (0.4%)	1 (0.3%)	
Survival status				0.3612‡
Alive	67 (66.3%)	160 (68.4%)	227 (67.8%)	
Dead	33 (32.7%)	60 (25.6%)	93 (27.8%)	
NA	1 (1%)	14 (6%)	15 (4.5%)	

NA, not applicable.

^†^Wilcoxon rank sum test.

^‡^Pearson's *χ*^2^-test.
