# Supplementary material for: Frequent alterations in cytoskeleton remodelling genes in primary and metastatic lung adenocarcinomas
Source: Nat Commun. 2015 Dec 9;6:10131. doi: 10.1038/ncomms10131 (PMC4682110; doi:10.1038/ncomms10131)

Supplementary figures

Supplementary figure 1

Pathology- Hematoxylin and Eosin Staining of lung adenocarcinoma tissues: a), b), c) showed histologic patterns of primary tumor at 100X, 200X and 400X; d), e), f) showed histologic patterns of corresponding lymph node metastasis at 100X, 200X and 400X. Scale bars represent 100µm.

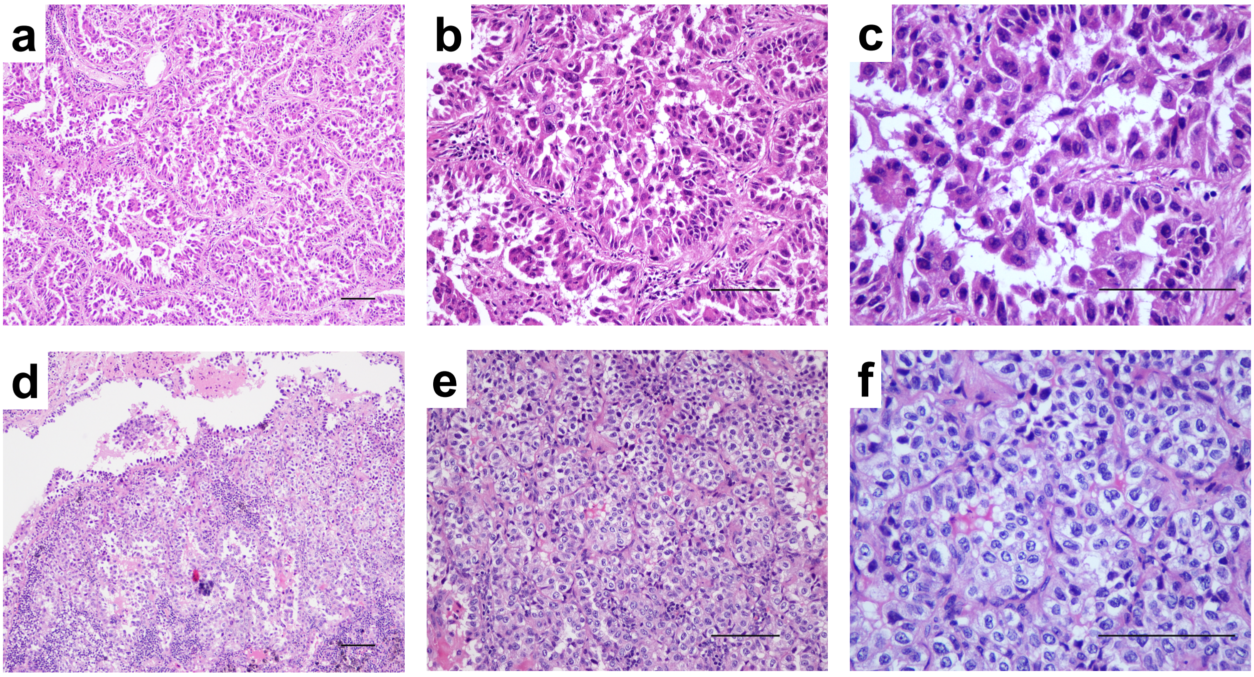

Supplementary figure 2

Hierarchical clustering of 101 primary lung adenocarcinomas according to the nucleotide context-specific exonic mutation rates. Top bar: Gender, smoking status and tumor stage of each patient. Left bar: specific single nucleotide context mutational signature.

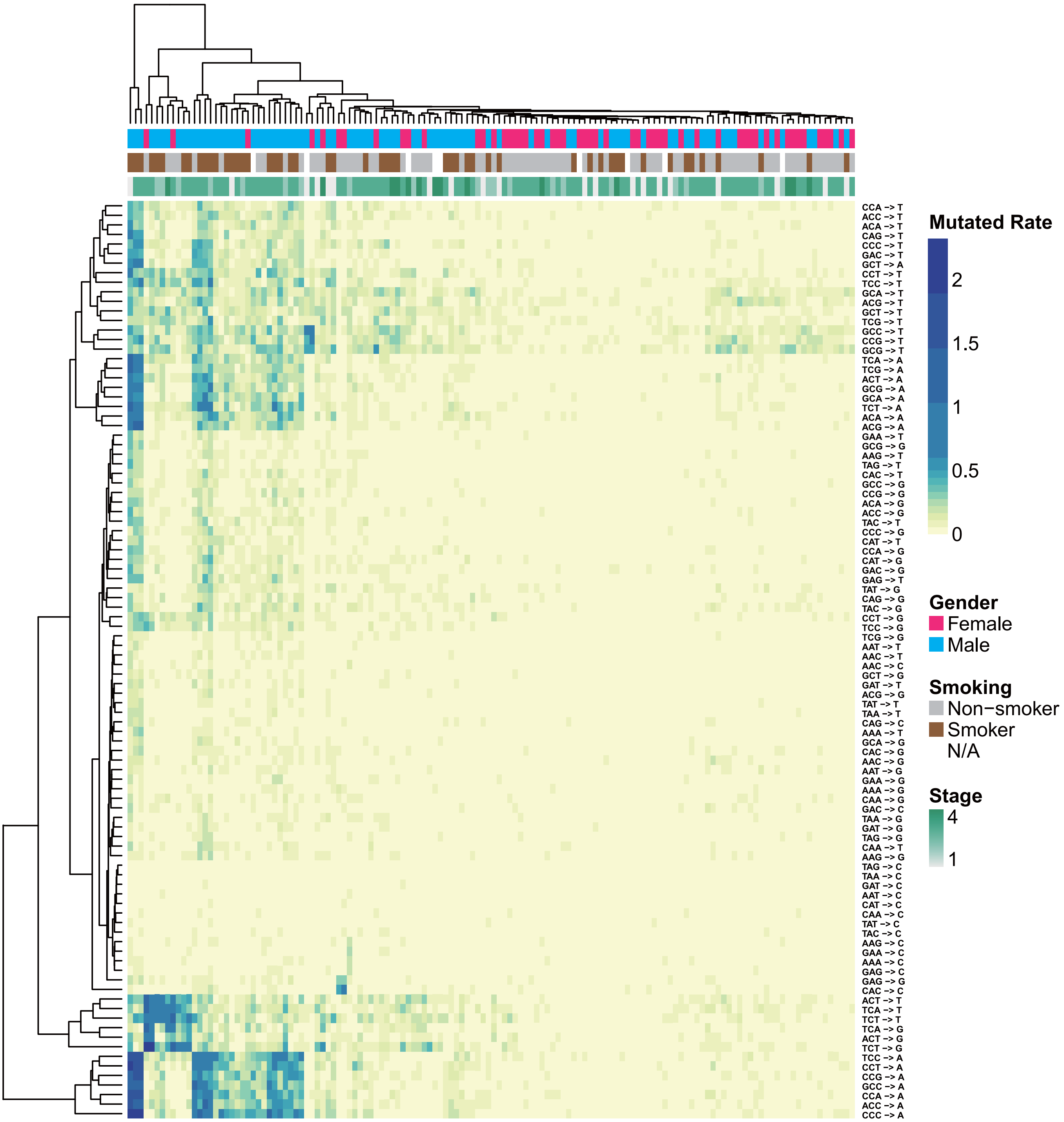

Supplementary figure 3

mRNA expression levels of APOBEC enzymes were profiled in primary tumors, metastatic tumors and adjacent normal tissues from 24 patients with whole genome and transcriptome sequencing data. Related to Figure 1 and Supplementary figure 4.

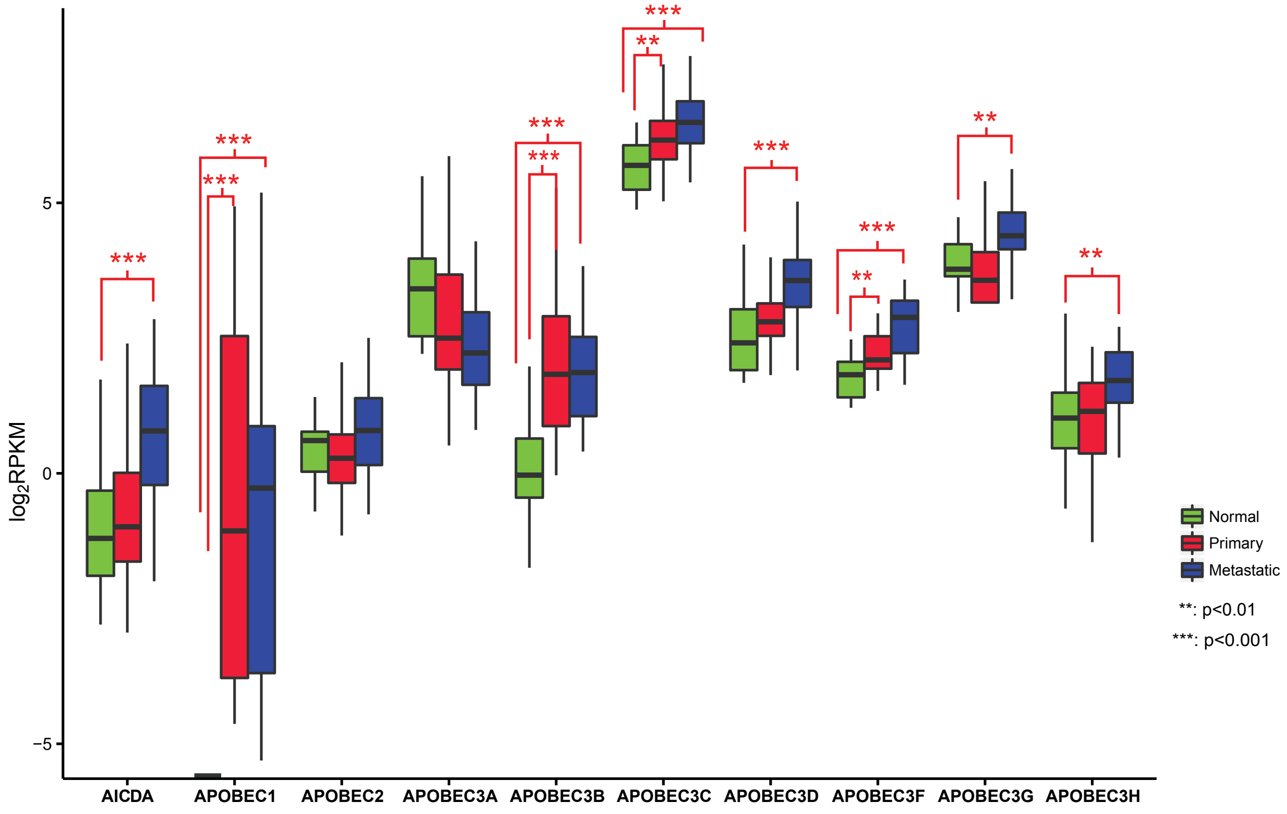

Supplementary figure 4

Mutational signatures of lung adenocarcinoma, Related to Figure 1: a) Association of signature contribution and proportion of indel mutation. b) Signature 2 contribution in primary lung adenocarcinomas in this study and lung adenocarcinomas derived from Europeans.

a.

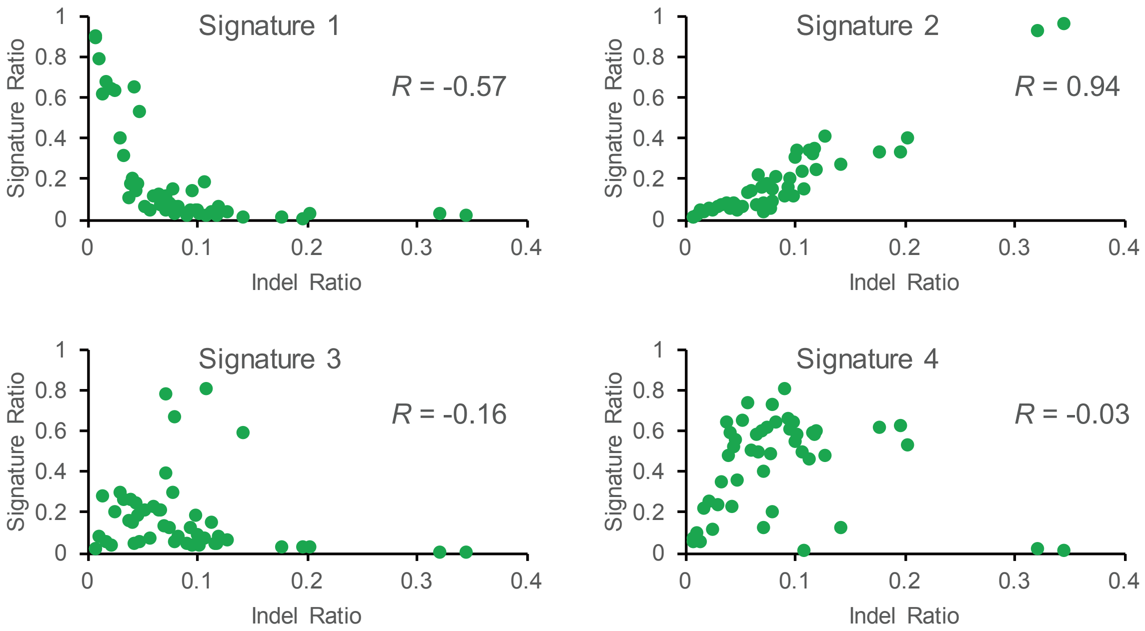

b.

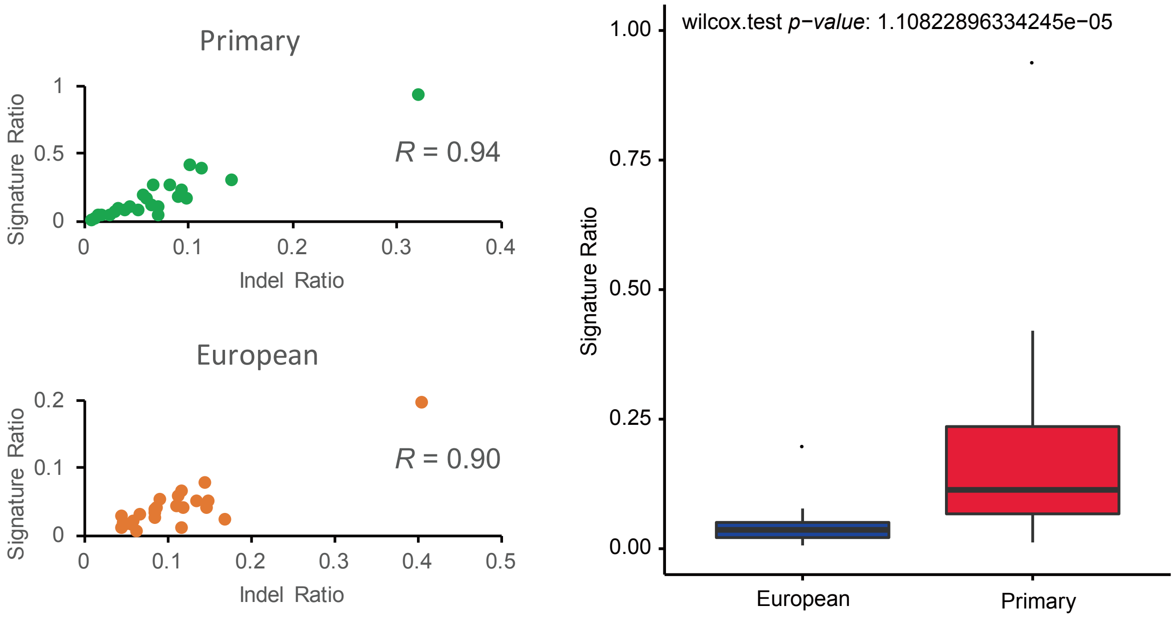

Supplementary figure 5

Mutational signatures of lung adenocarcinoma, Related to Figure 1: a) Contributions of mutational signatures to each individual tumor. b) Correlation of the contributions of mutational signatures between paired primary and metastatic tumors.

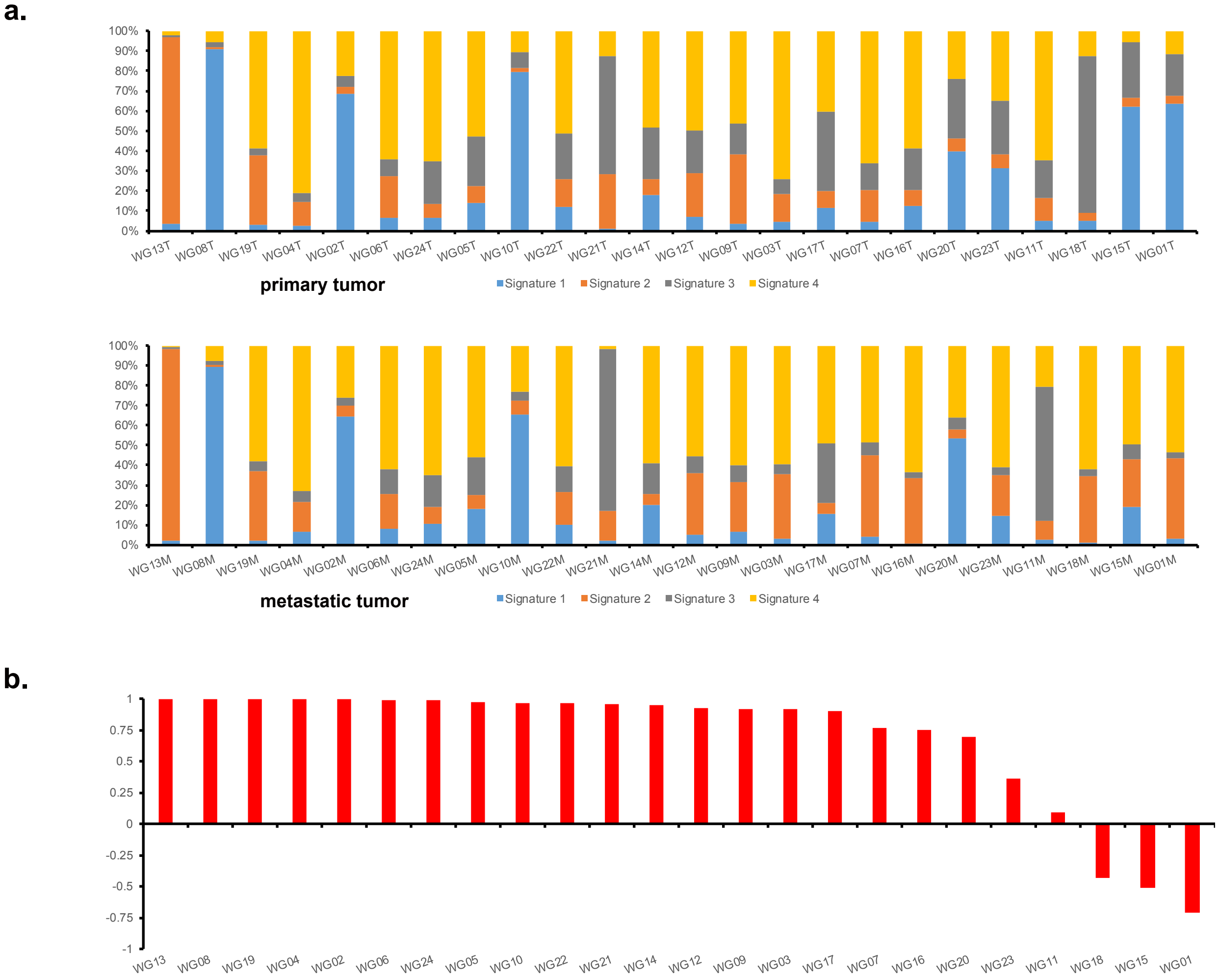

Supplementary figure 6

Somatic mutations in recurrently mutated genes: a) Constitution of synonymous and non-synonymous mutations of each mutated gene. b) Mutation types and relative positions of somatic mutations are shown (genorated by MutationMapper, http://www.cbioportal.org/mutation_mapper.jsp) in the transcript of each mutated gene.

a.

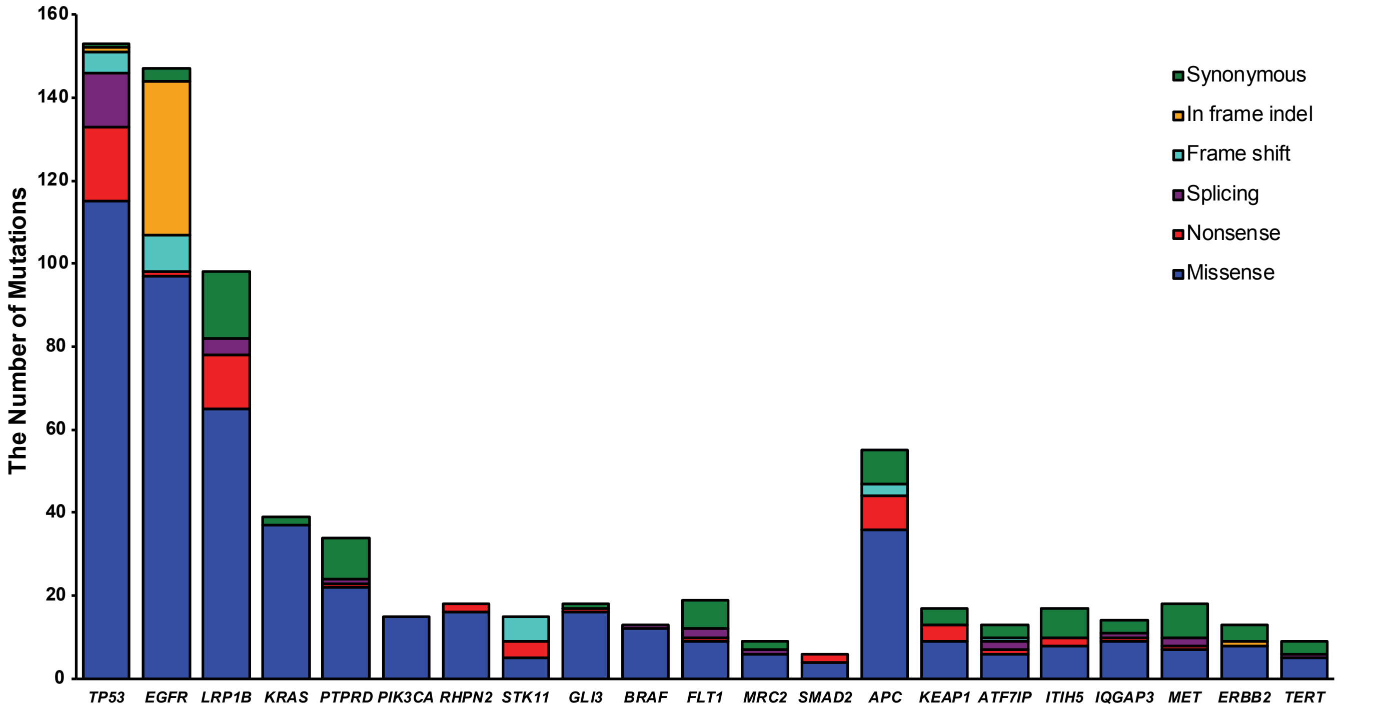

b.

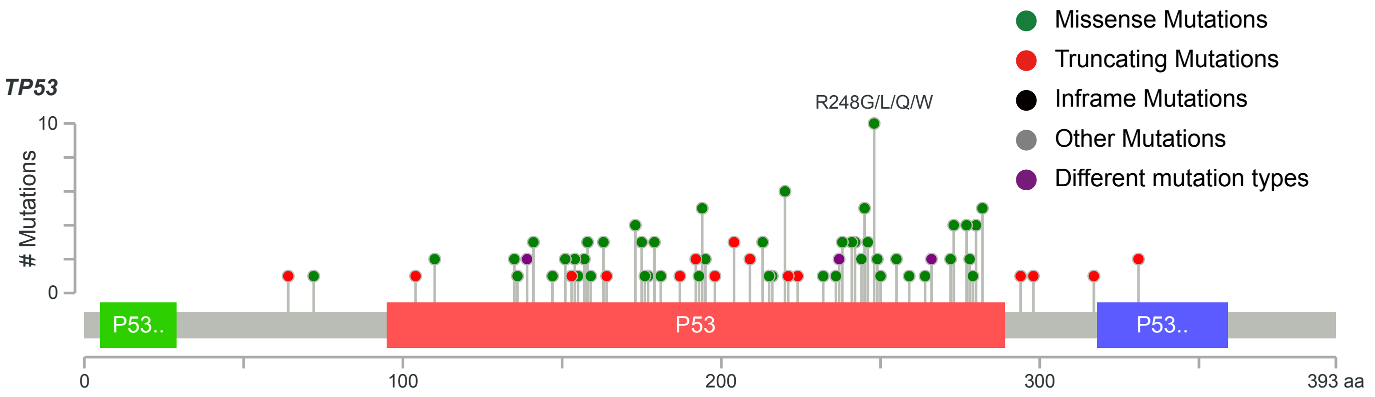

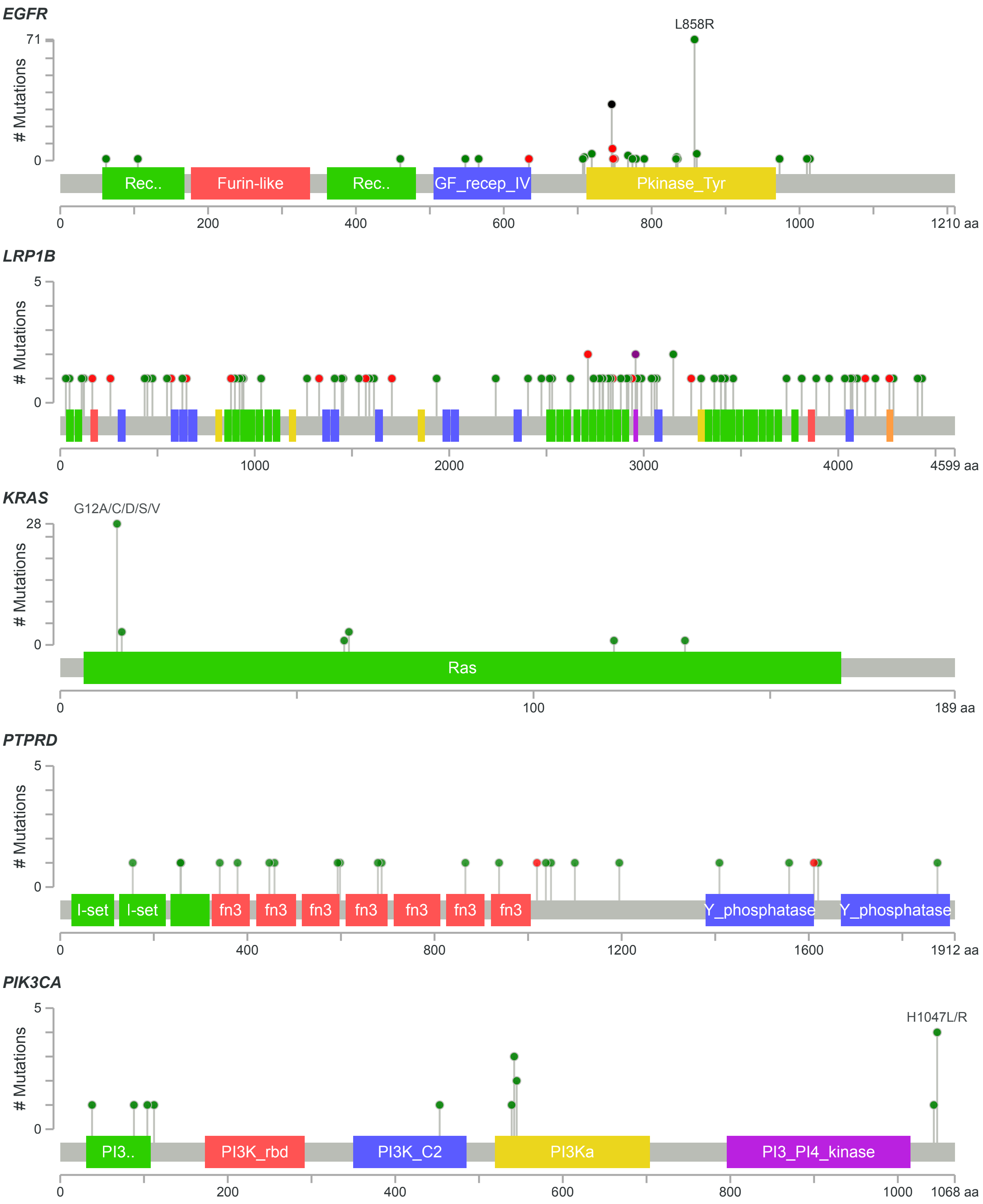
EGFR
L858R
71
0
Mutations
Rec..
Furin-like
Rec..
GF_recep_IV
Pkinase_Tyr
0
200
400
600
800
1000
1210 aa
LRP1B
5
0
Mutations
0
1000
2000
3000
4000
4599 aa
KRAS
G12A/C/D/S/V
28
0
Mutations
Ras
0
100
189 aa
PTPRD
5
0
Mutations
I-set
I-set
fn3
fn3
fn3
fn3
fn3
fn3
fn3
Y_phosphatase
Y_phosphatase
0
400
800
1200
1600
1912 aa
PIK3CA
H1047L/R
5
0
Mutations
PI3..
PI3K_rbd
PI3K_C2
PI3Ka
PI3_PI4_kinase
0
200
400
600
800
1000
1068 aa

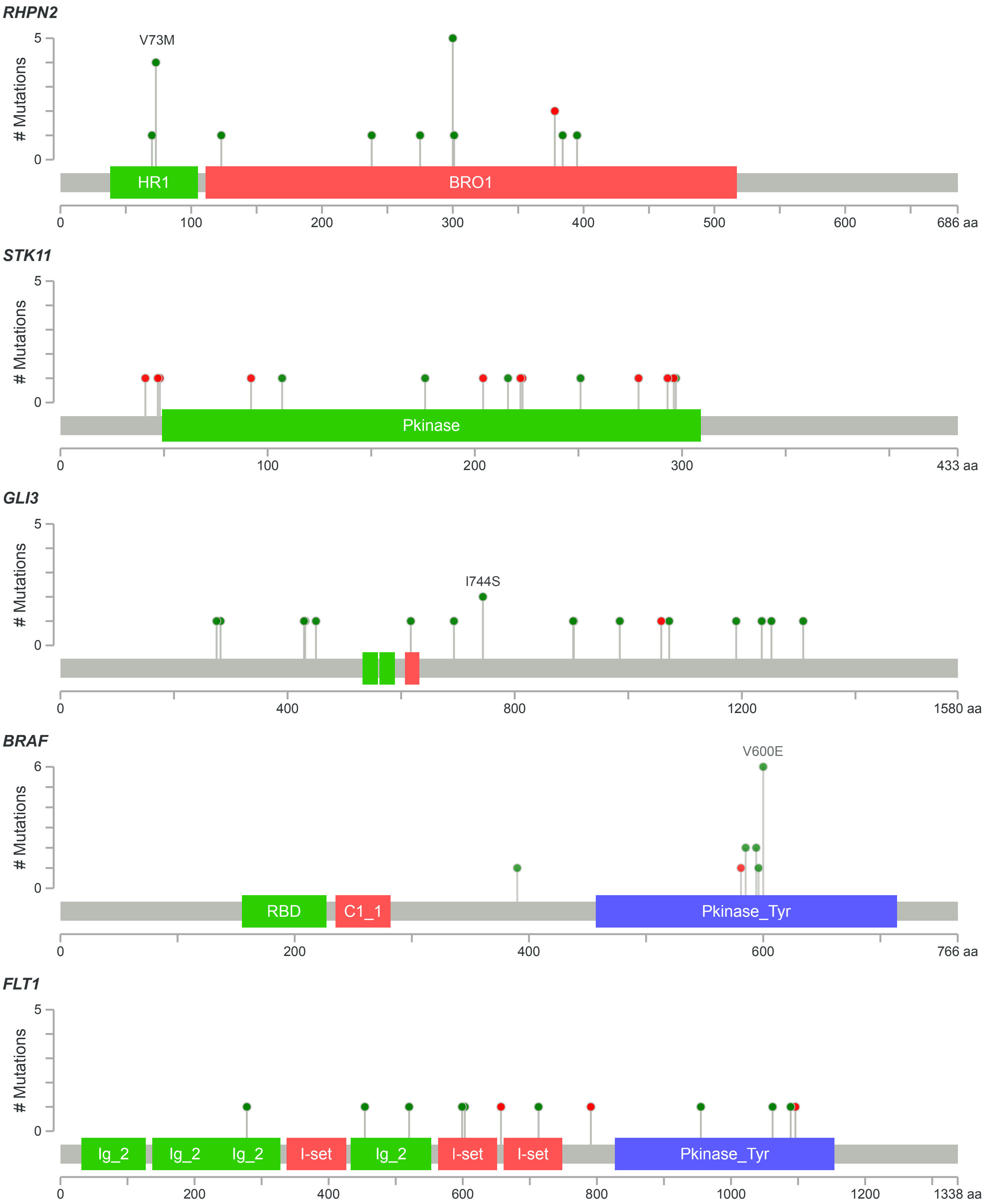

RHPN2
Mutations
0
5
V73M
HR1
BRO1
0
100
200
300
400
500
600
686 aa
STK11
Mutations
0
5
Pkinase
0
100
200
300
433 aa
GLI3
Mutations
0
5
I744S
0
400
800
1200
1580 aa
BRAF
Mutations
0
6
V600E
RBD
C1_1
Pkinase_Tyr
0
200
400
600
766 aa
FLT1
Mutations
0
5
Ig_2
Ig_2
Ig_2
I-set
Ig_2
I-set
I-set
Pkinase_Tyr
0
200
400
600
800
1000
1200
1338 aa

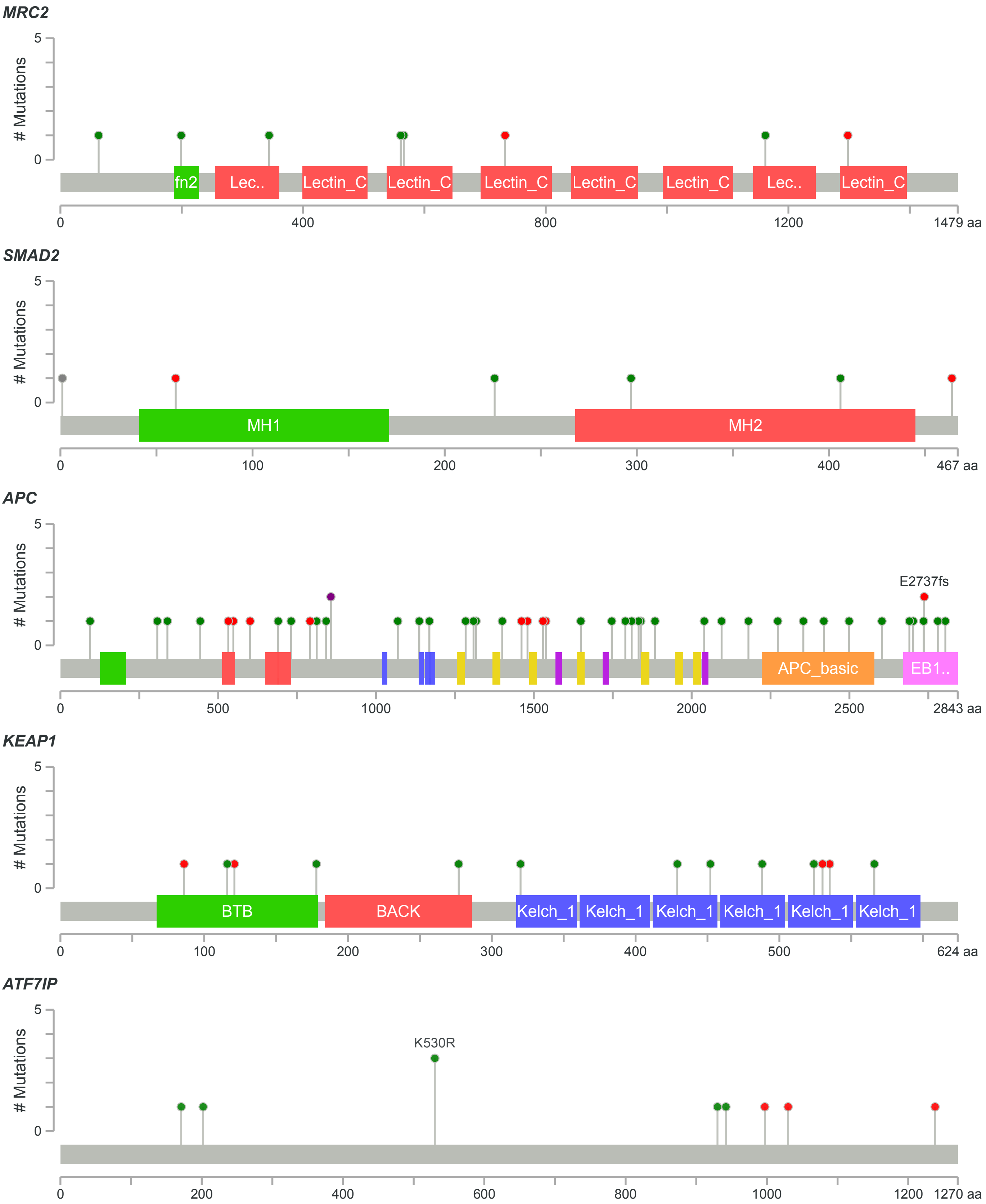
MRC2
5
0
Mutations
fn2
Lec..
Lectin_C
Lectin_C
Lectin_C
Lectin_C
Lectin_C
Lec..
Lectin_C
0
400
800
1200
1479 aa
SMAD2
5
0
Mutations
MH1
MH2
0
100
200
300
400
467 aa
APC
5
0
Mutations
E2737fs
APC_basic
EB1..
0
500
1000
1500
2000
2500
2843 aa
KEAP1
5
0
Mutations
BTB
BACK
Kelch_1
Kelch_1
Kelch_1
Kelch_1
Kelch_1
Kelch_1
0
100
200
300
400
500
624 aa
ATF7IP
5
0
Mutations
K530R
0
200
400
600
800
1000
1200
1270 aa

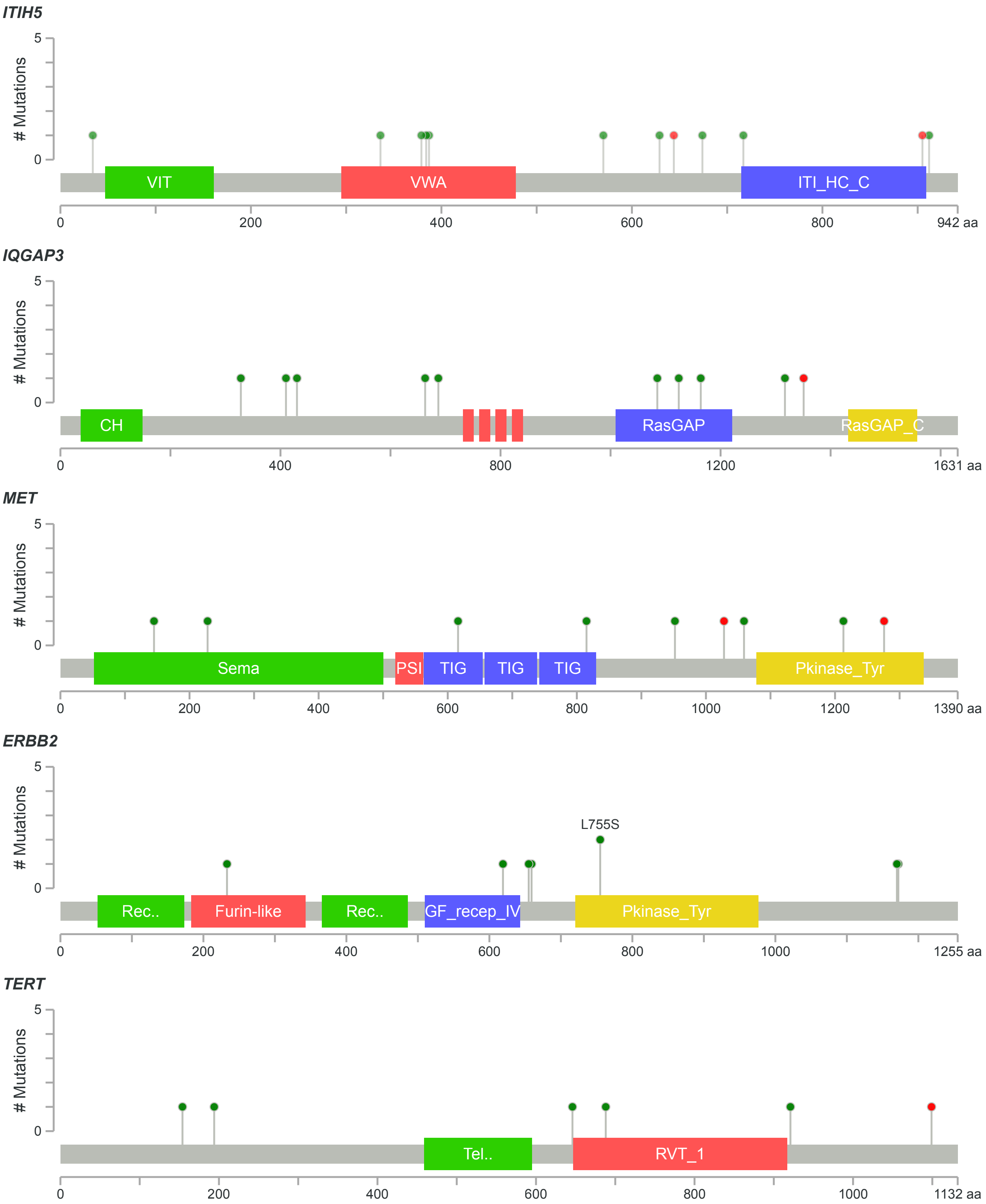
ITIH5
Mutations
5
0
VIT
VWA
ITI_HC_C
0
200
400
600
800
942 aa
IQGAP3
Mutations
5
0
CH
RasGAP
RasGAP_C
0
400
800
1200
1631 aa
MET
Mutations
5
0
Sema
PSI
TIG
TIG
TIG
Pkinase_Tyr
0
200
400
600
800
1000
1200
1390 aa
ERBB2
Mutations
5
0
L755S
Rec..
Furin-like
Rec..
GF_recep_IV
Pkinase_Tyr
0
200
400
600
800
1000
1255 aa
TERT
Mutations
5
0
Tel..
RVT_1
0
200
400
600
800
1000
1132 aa

Supplementary figure 7

Clinical association of mutated genes in 335 Chinese lung adenocarcinomas. Kaplan-Meier survival analysis was performed to determine the different overall survivals between patients with or without somatic mutations in specific genes.

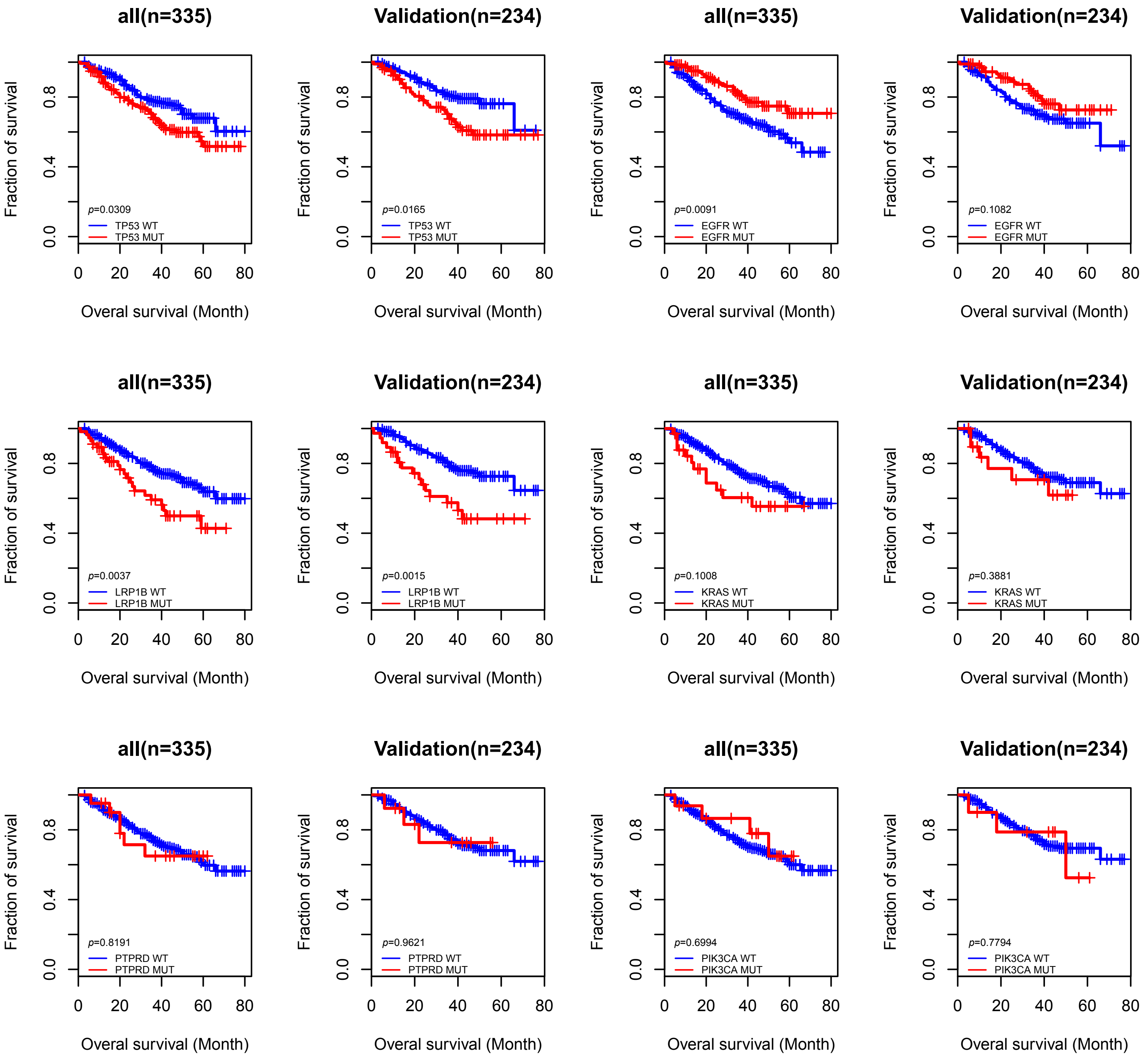

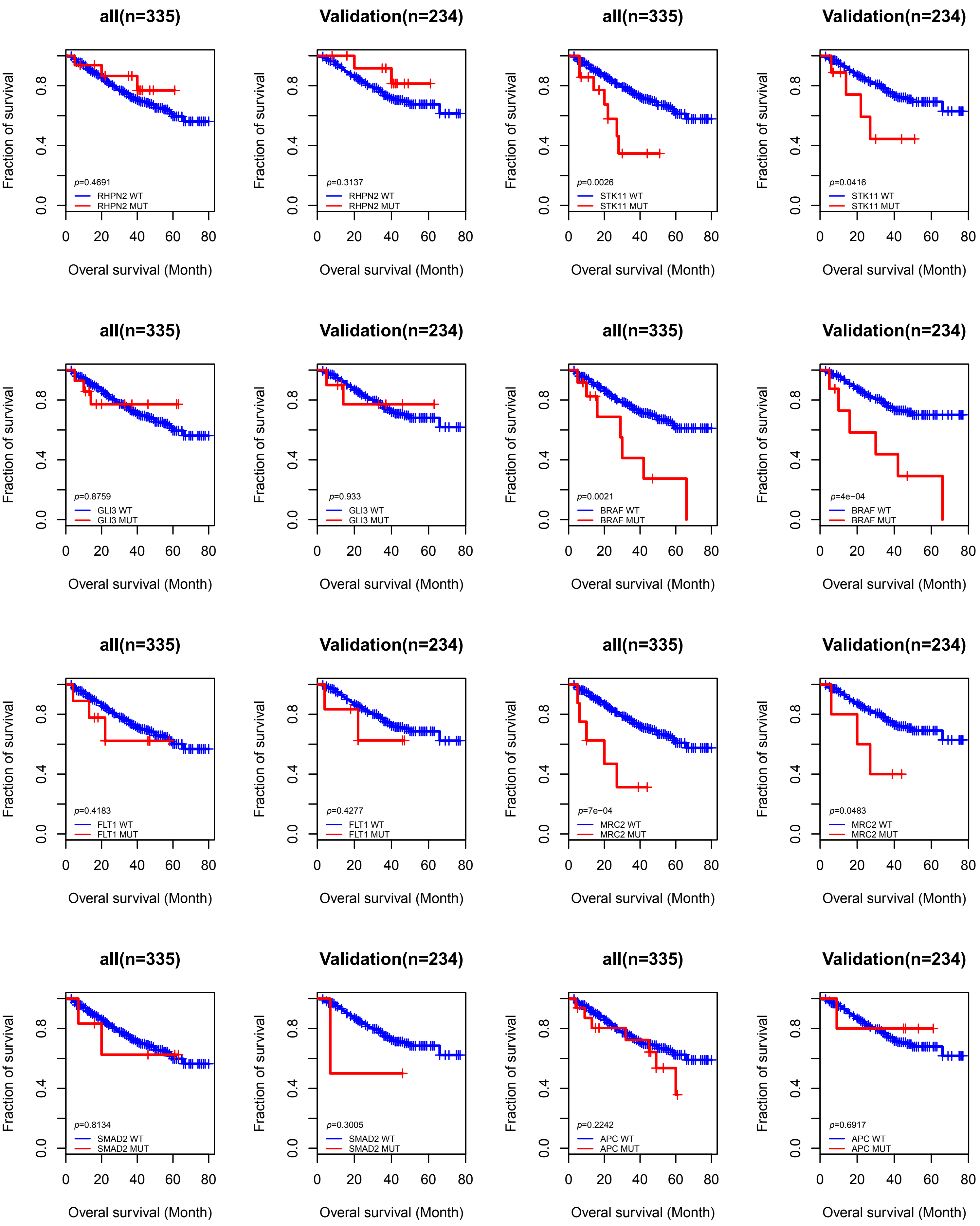
all(n=335)
Validation(n=234)
Fraction of survival
Overal survival (Month)
p=0.4691
RHPN2 WT
RHPN2 MUT
p=0.3137
RHPN2 WT
RHPN2 MUT
p=0.0026
STK11 WT
STK11 MUT
p=0.0416
STK11 WT
STK11 MUT
p=0.8759
GLI3 WT
GLI3 MUT
p=0.933
GLI3 WT
GLI3 MUT
p=0.0021
BRAF WT
BRAF MUT
p=4e−04
BRAF WT
BRAF MUT
p=0.4183
FLT1 WT
FLT1 MUT
p=0.4277
FLT1 WT
FLT1 MUT
p=7e−04
MRC2 WT
MRC2 MUT
p=0.0483
MRC2 WT
MRC2 MUT
p=0.8134
SMAD2 WT
SMAD2 MUT
p=0.3005
SMAD2 WT
SMAD2 MUT
p=0.2242
APC WT
APC MUT
p=0.6917
APC WT
APC MUT
0.0
0.4
0.8
0
20
40
60
80

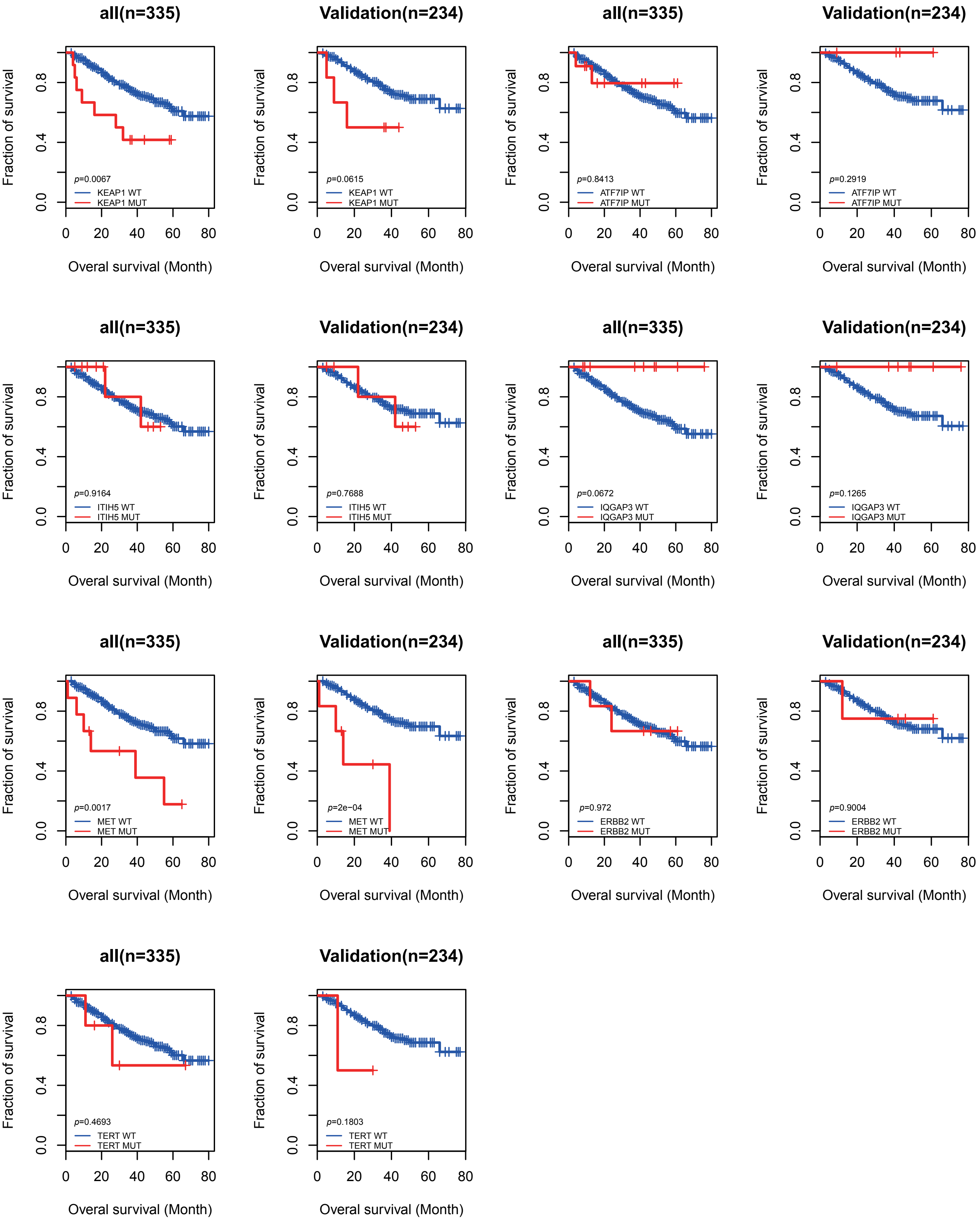
all(n=335)
Validation(n=234)
Fraction of survival
Overal survival (Month)
0.0
0.4
0.8
0
20
40
60
80
p=0.0067
KEAP1 WT
KEAP1 MUT
p=0.0615
KEAP1 WT
KEAP1 MUT
p=0.8413
ATF7IP WT
ATF7IP MUT
p=0.2919
ATF7IP WT
ATF7IP MUT
p=0.9164
ITIH5 WT
ITIH5 MUT
p=0.7688
ITIH5 WT
ITIH5 MUT
p=0.0672
IQGAP3 WT
IQGAP3 MUT
p=0.1265
IQGAP3 WT
IQGAP3 MUT
p=0.0017
MET WT
MET MUT
p=2e−04
MET WT
MET MUT
p=0.972
ERBB2 WT
ERBB2 MUT
p=0.9004
ERBB2 WT
ERBB2 MUT
p=0.4693
TERT WT
TERT MUT
p=0.1803
TERT WT
TERT MUT

Supplementary figure 8

Frequency of broad copy number alterations (CNAs) across 101 primary lung adenocarcinomas and 35 metastatic tumors. Chromosomal arm regions with copy gain (red) and loss (blue) were shown.

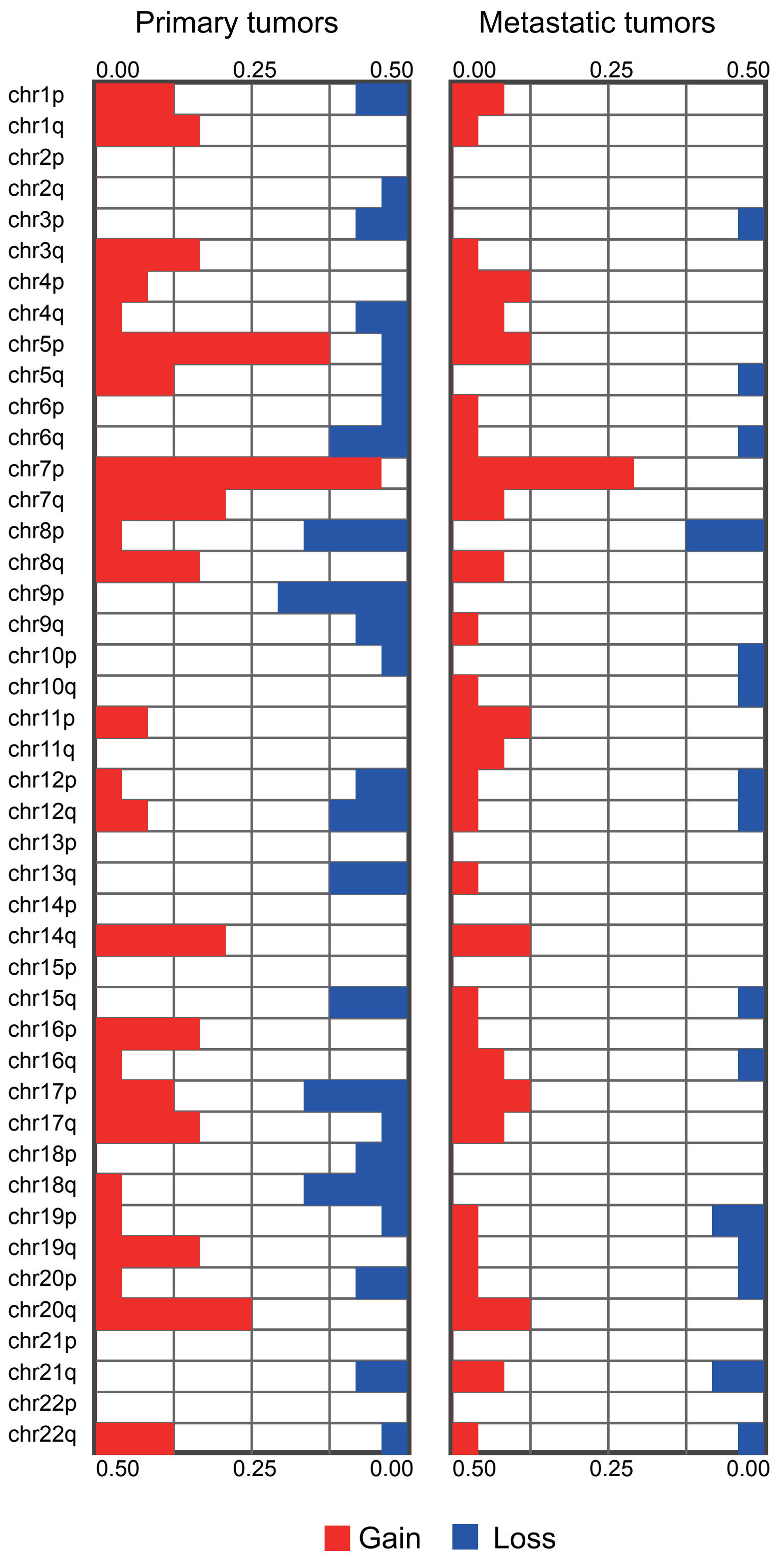

Supplementary figure 9

Correlations of somatic copy number alterations and corresponding mRNA expression status of affected genes. Abscissa shows G-score of copy number change for each altered gene, ordinate shows log2 expression fold change of genes altered by CNV. Each dot in the figure represents a gene affected by SCNA.

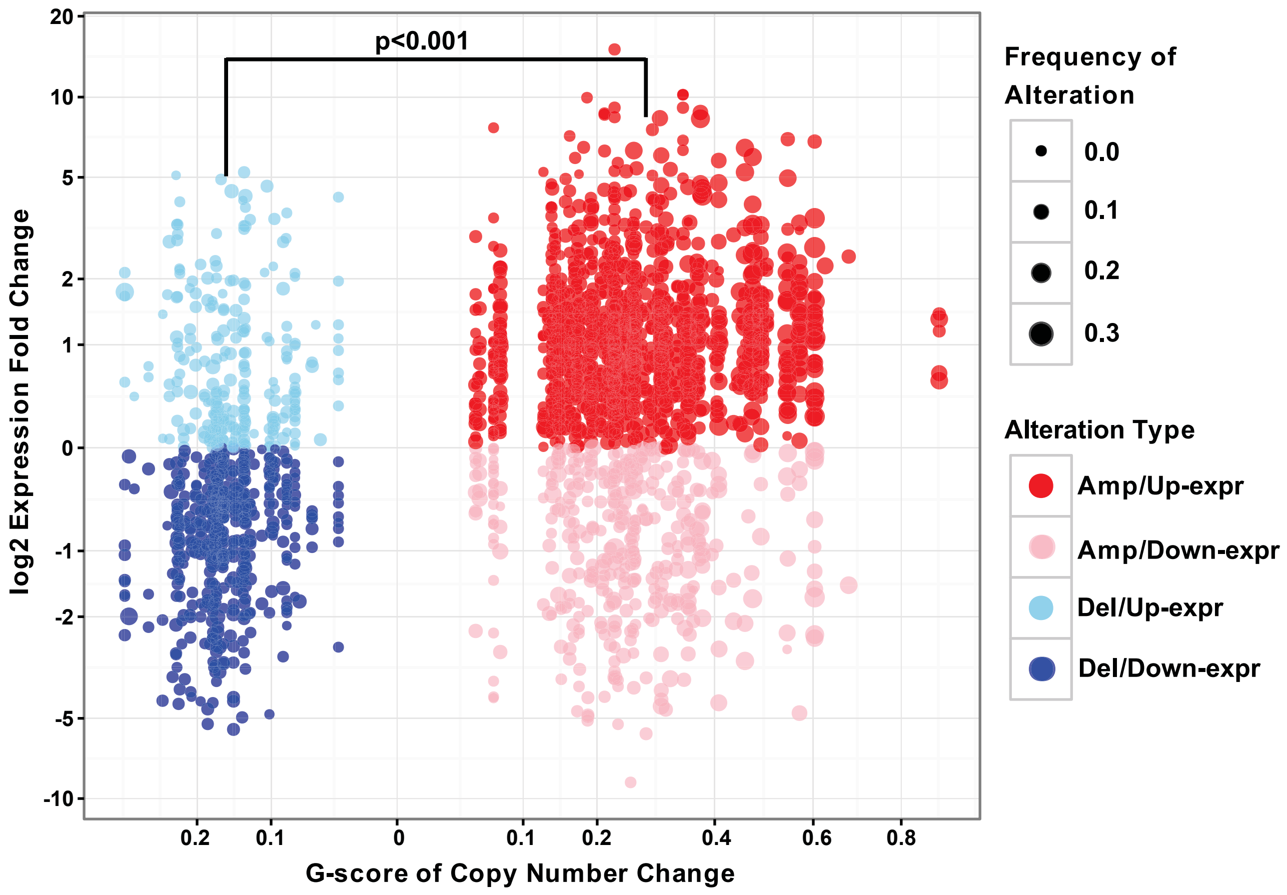

Supplementary figure 10

Kaplan-Meier survival curve showed that patients with transcriptional pattern Cluster 3 had significantly shorter overall survival than those harboring transcriptional pattern clusters 1 and 2.

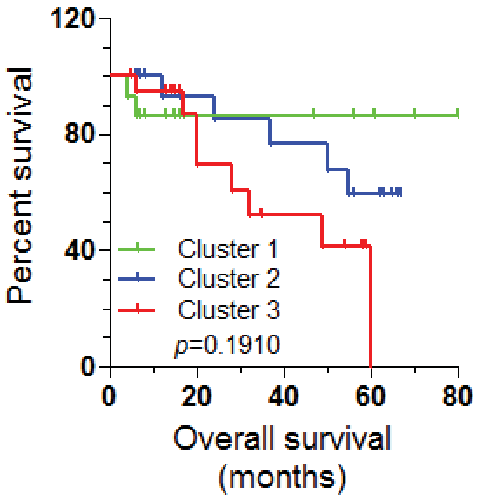

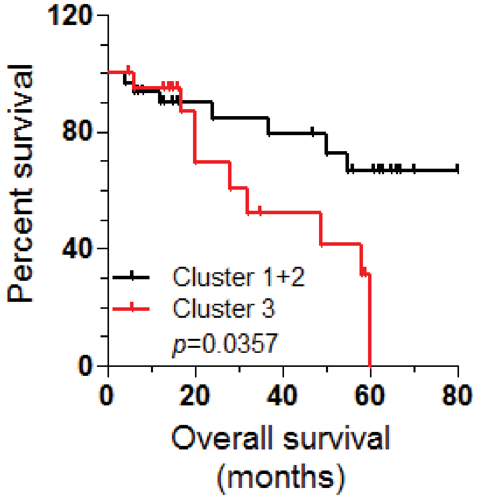

Supplementary figure 11

CIRCOS Plots of whole-genome sequence data from 24 primary lung adenocarcinomas and corresponding lymph node metastases. The outer ring shows the karyotype of the human genome, and the inner ring displays copy number alterations identified from WGS, with amplifications in red and deletions in blue. Genomic rearrangements are also shown with inter-chromosomal events in purple and intra-chromosomal events in green.

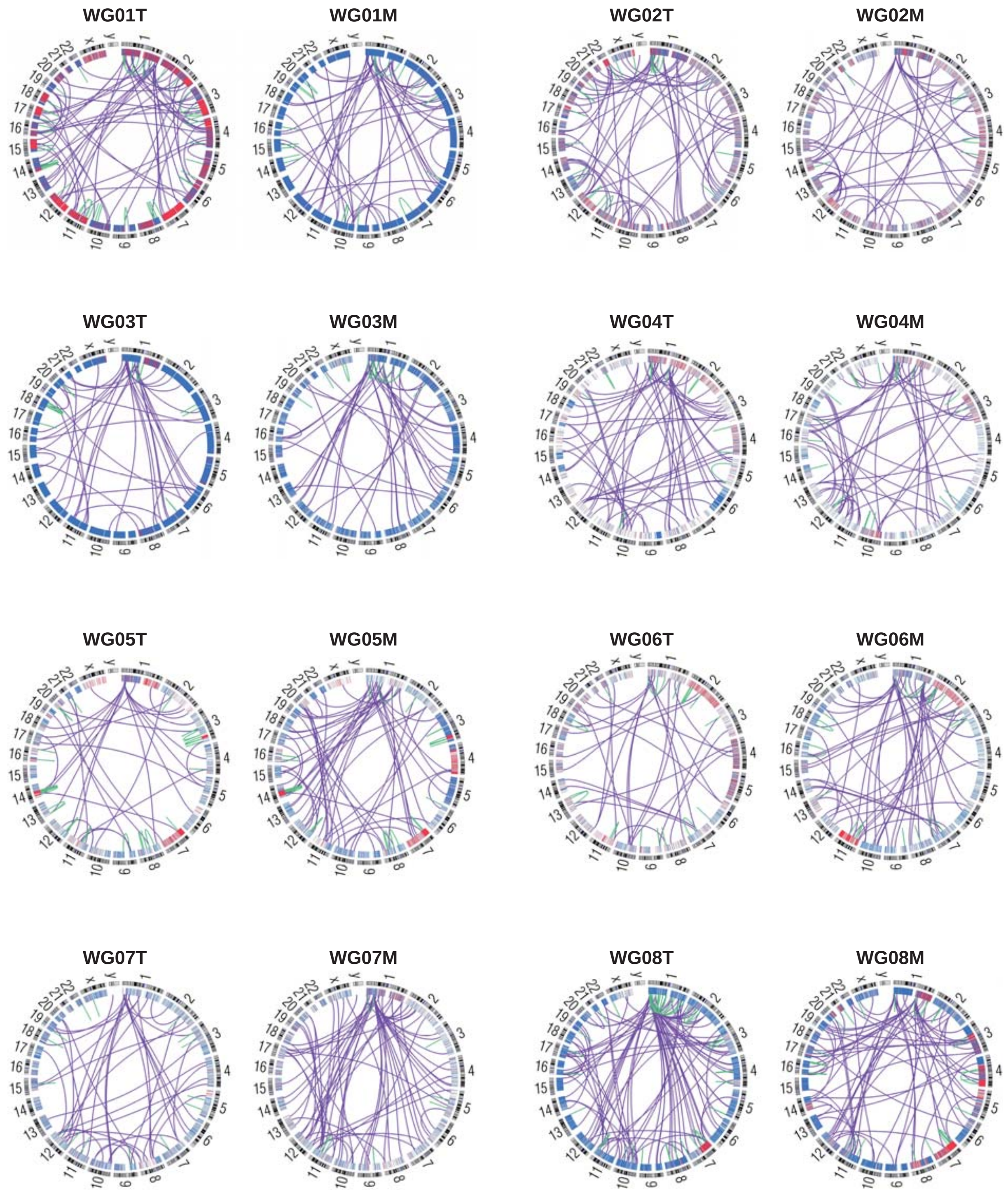

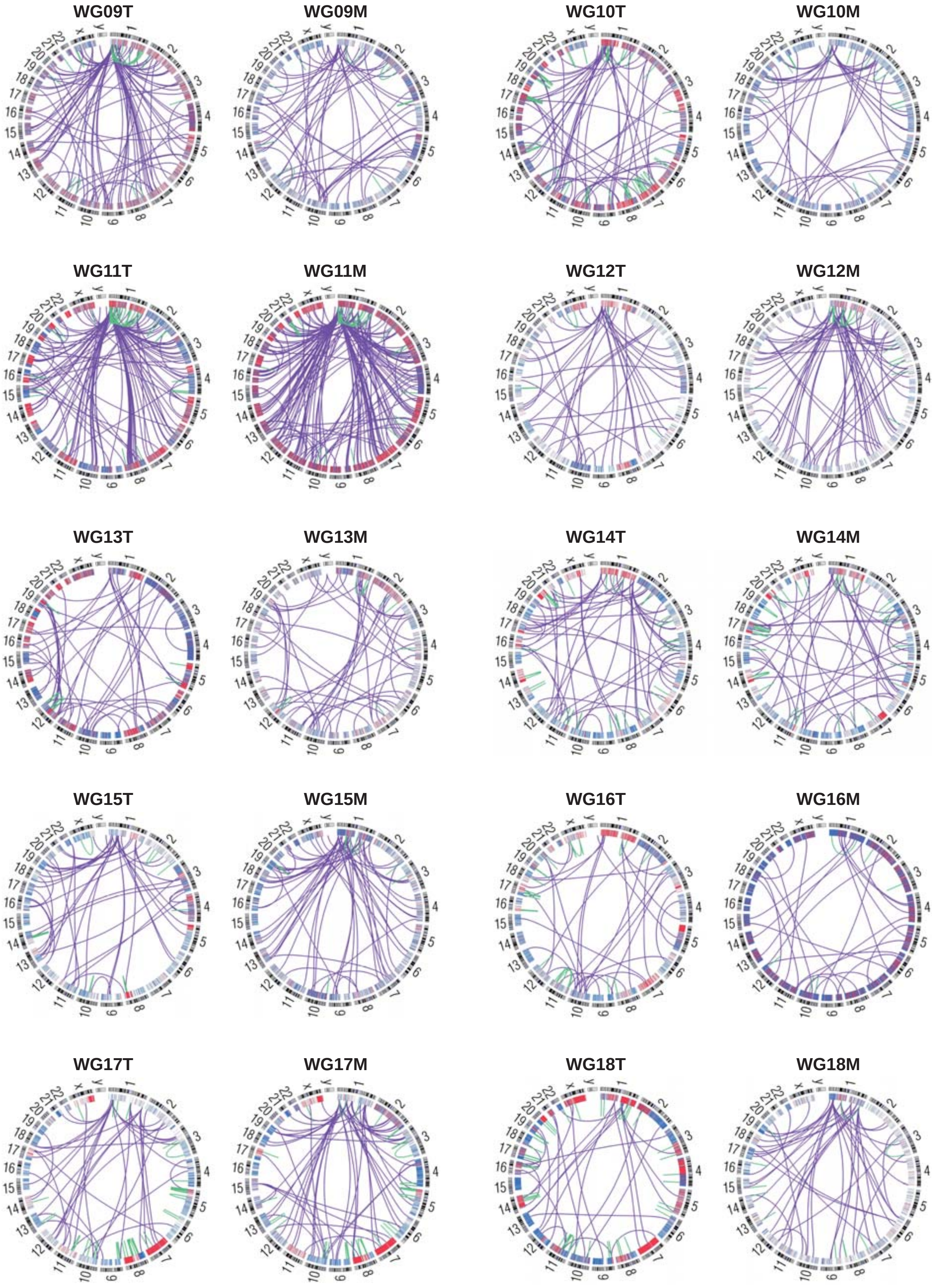
WG09T
WG09M
WG10T
WG10M
WG11T
WG11M
WG12T
WG12M
WG13T
WG13M
WG14T
WG14M
WG15T
WG15M
WG16T
WG16M
WG17T
WG17M
WG18T
WG18M

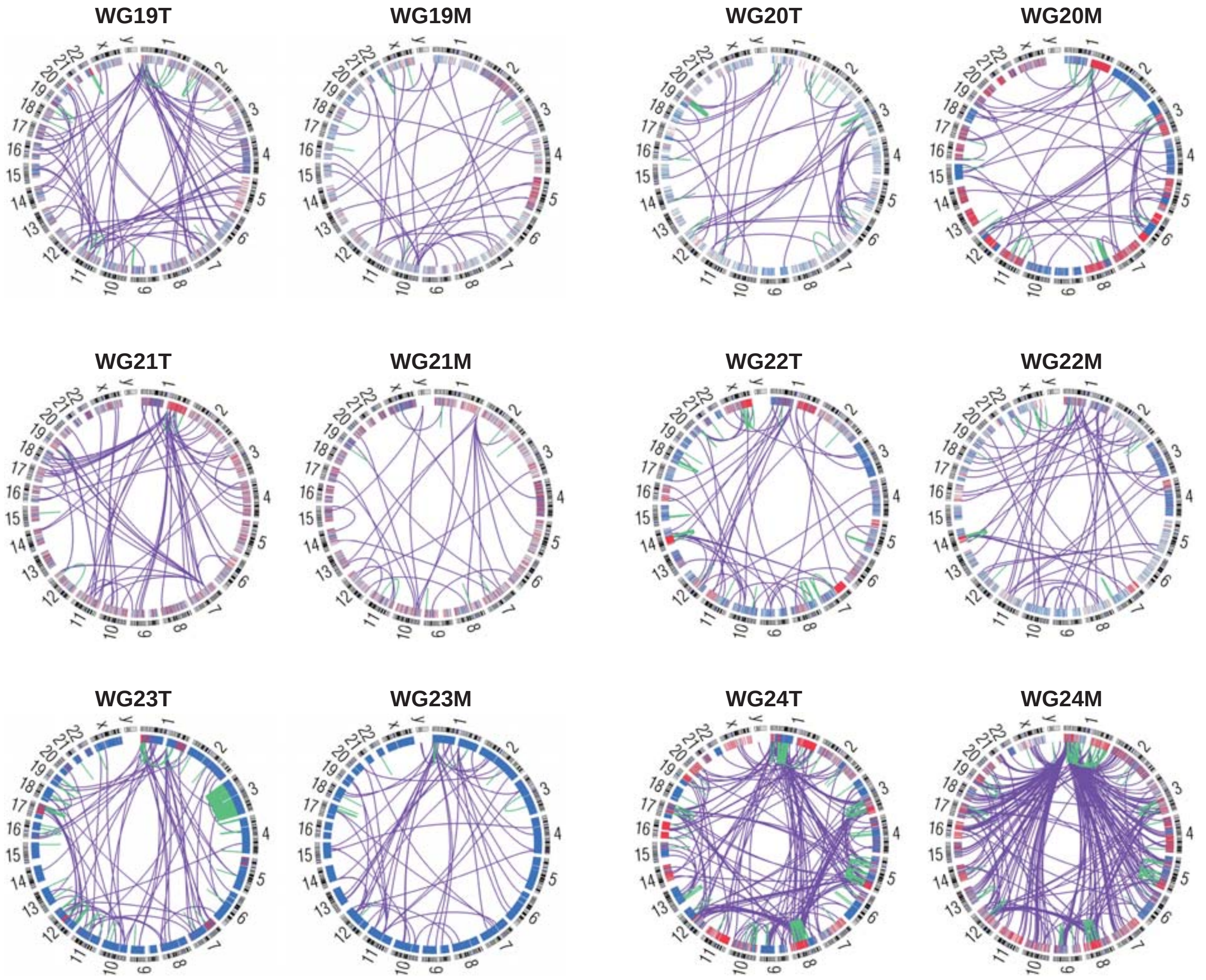
WG19T
WG19M
WG20T
WG20M
WG21T
WG21M
WG22T
WG22M
WG23T
WG23M
WG24T
WG24M

Supplementary figure 12

Concurrent and mutual exclusion analysis of genomic alterations and clinical features. Concurrence and mutual exclusion analysis was performed on significantly mutated genes, significant copy number variations and translocations or fusion events, as well as clinical features such as smoking, age and metastasis status. Significance was calculated by Fisher's exact test.

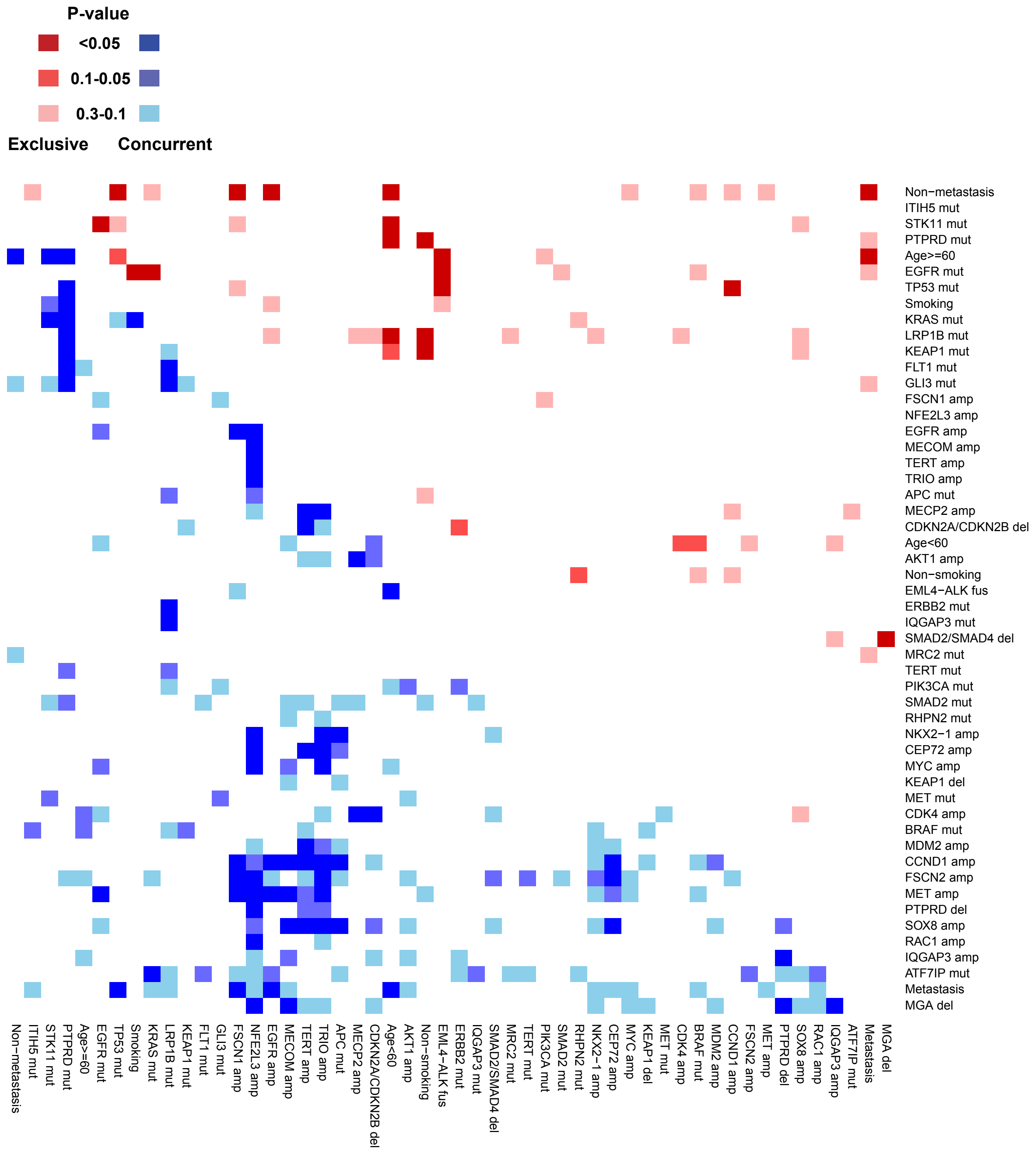

Supplementary figure 13

Gene-gene fusion events supported by both whole genome and transcriptome sequencing data. Figure shows the genomic structures and breakponits of 15 gene-gene fusions. Detailed information of the fusion regions and breakpoints was summarized in Supplementary table 15.

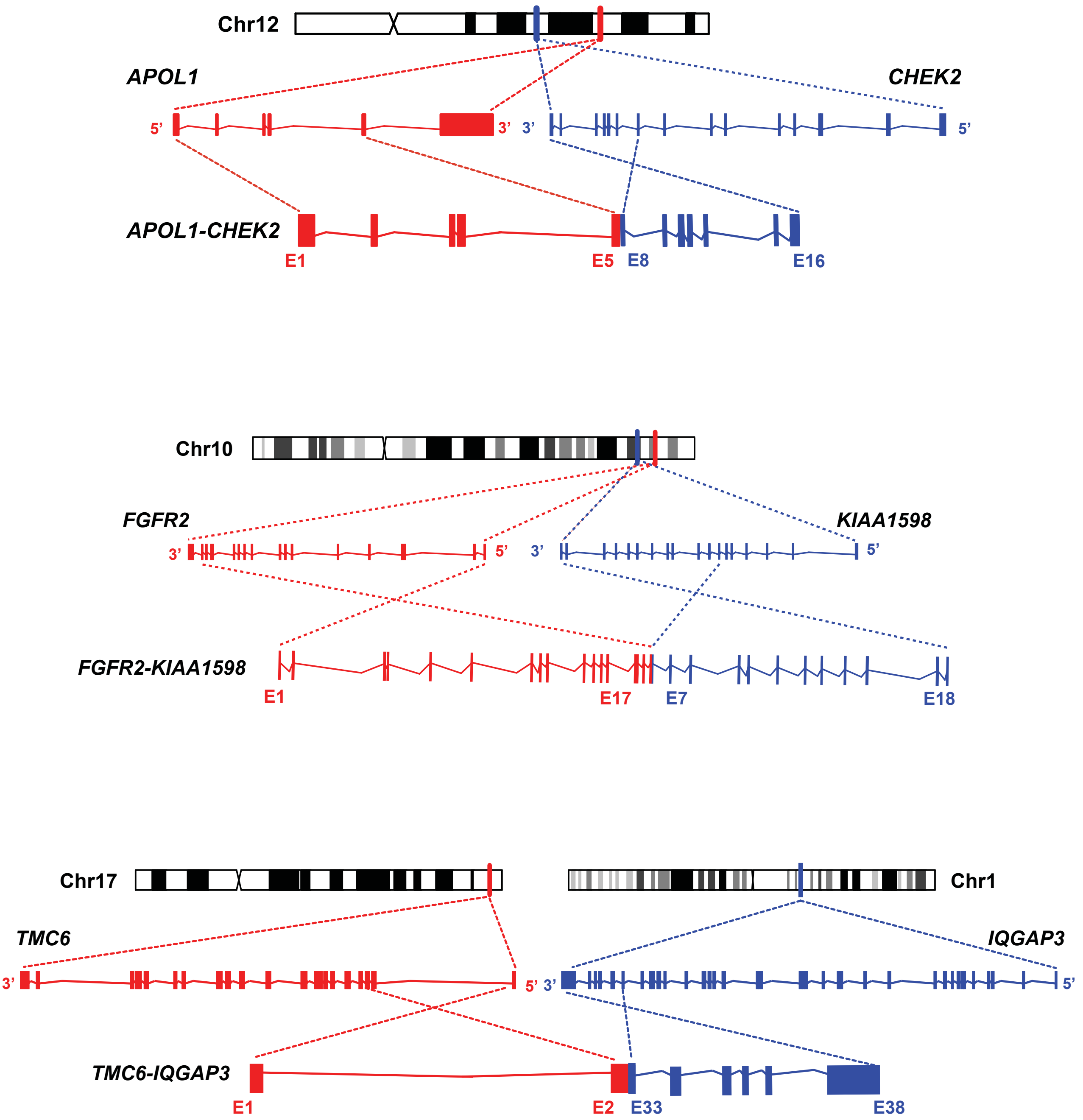

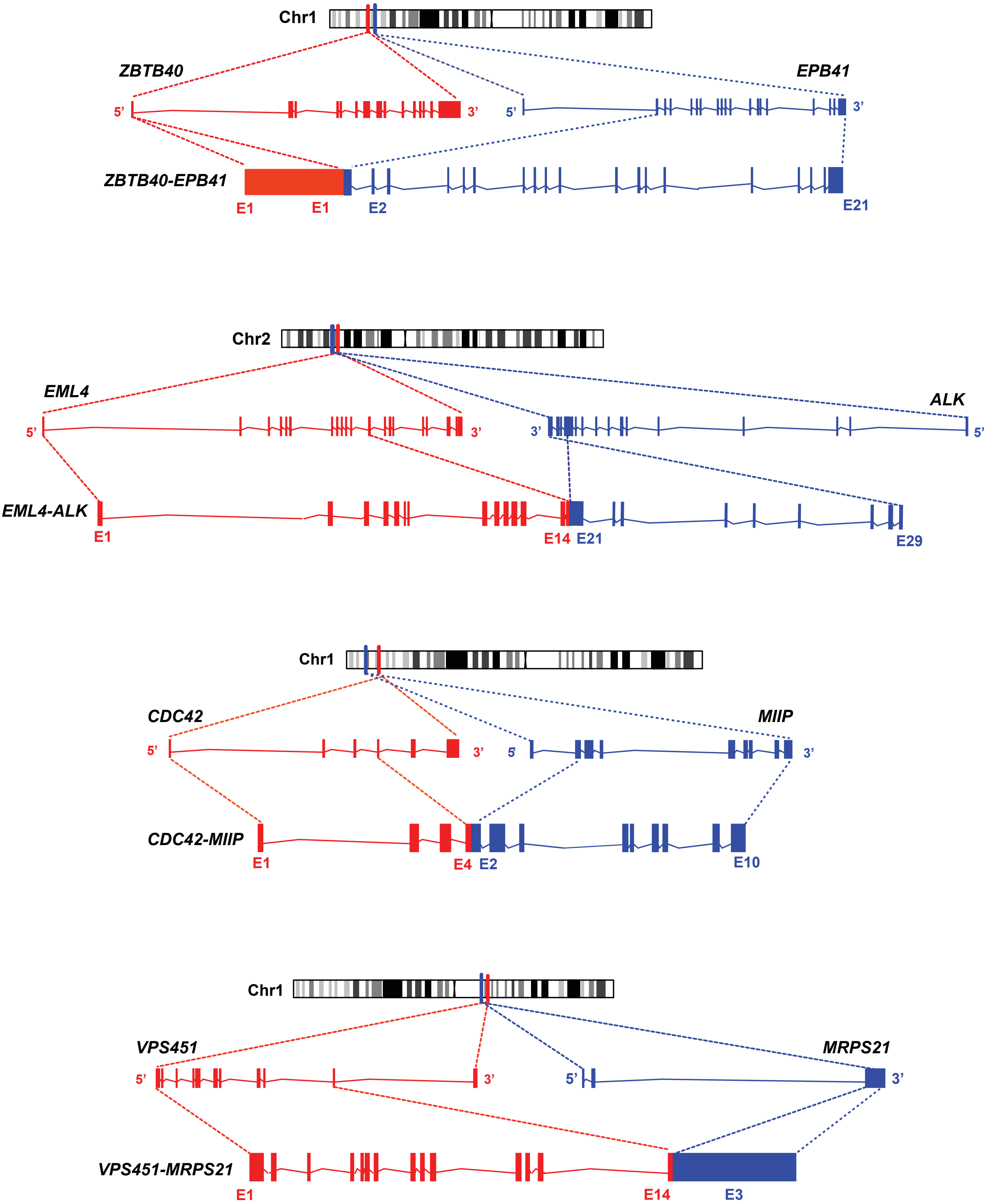

Chr1
ZBTB40
EPB41
5'
3'
5'
3'
ZBTB40-EPB41
E1
E1
E2
E21
Chr2
EML4
ALK
5'
3'
3'
5'
EML4-ALK
E1
E14
E21
E29
Chr1
CDC42
MIIP
5'
3'
5
3'
CDC42-MIIP
E1
E4
E2
E10
Chr1
VPS451
MRPS21
5'
3'
5'
3'
VPS451-MRPS21
E1
E14
E3

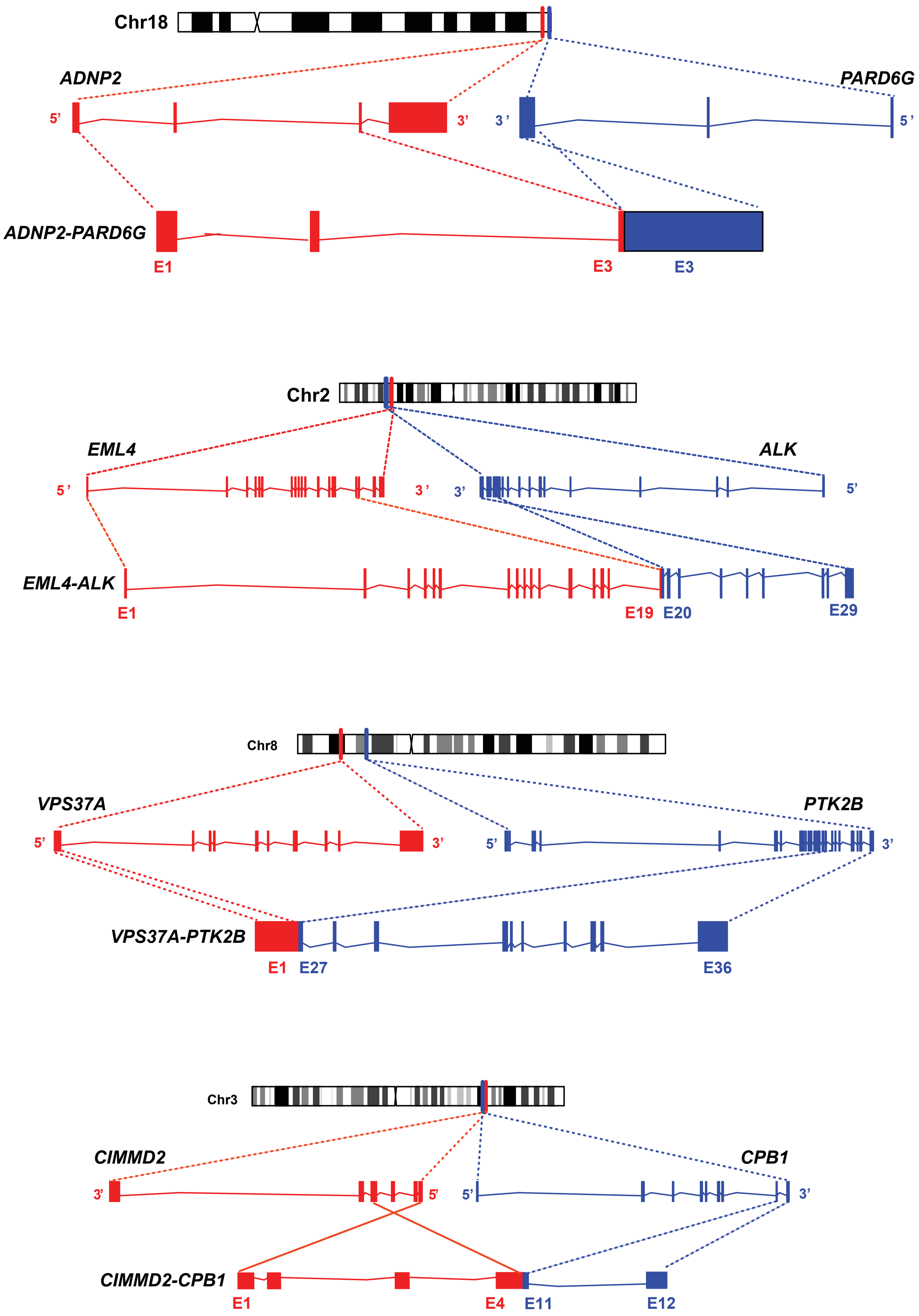

Chr18
ADNP2
PARD6G
5'
3'
3'
5'
ADNP2-PARD6G
E1
E3
E3
Chr2
EML4
ALK
5'
3'
3'
5'
EML4-ALK
E1
E19
E20
E29
Chr8
VPS37A
PTK2B
5'
3'
5'
3'
VPS37A-PTK2B
E1
E27
E36
Chr3
CIMMD2
CPB1
3'
5'
5'
3'
CIMMD2-CPB1
E1
E4
E11
E12

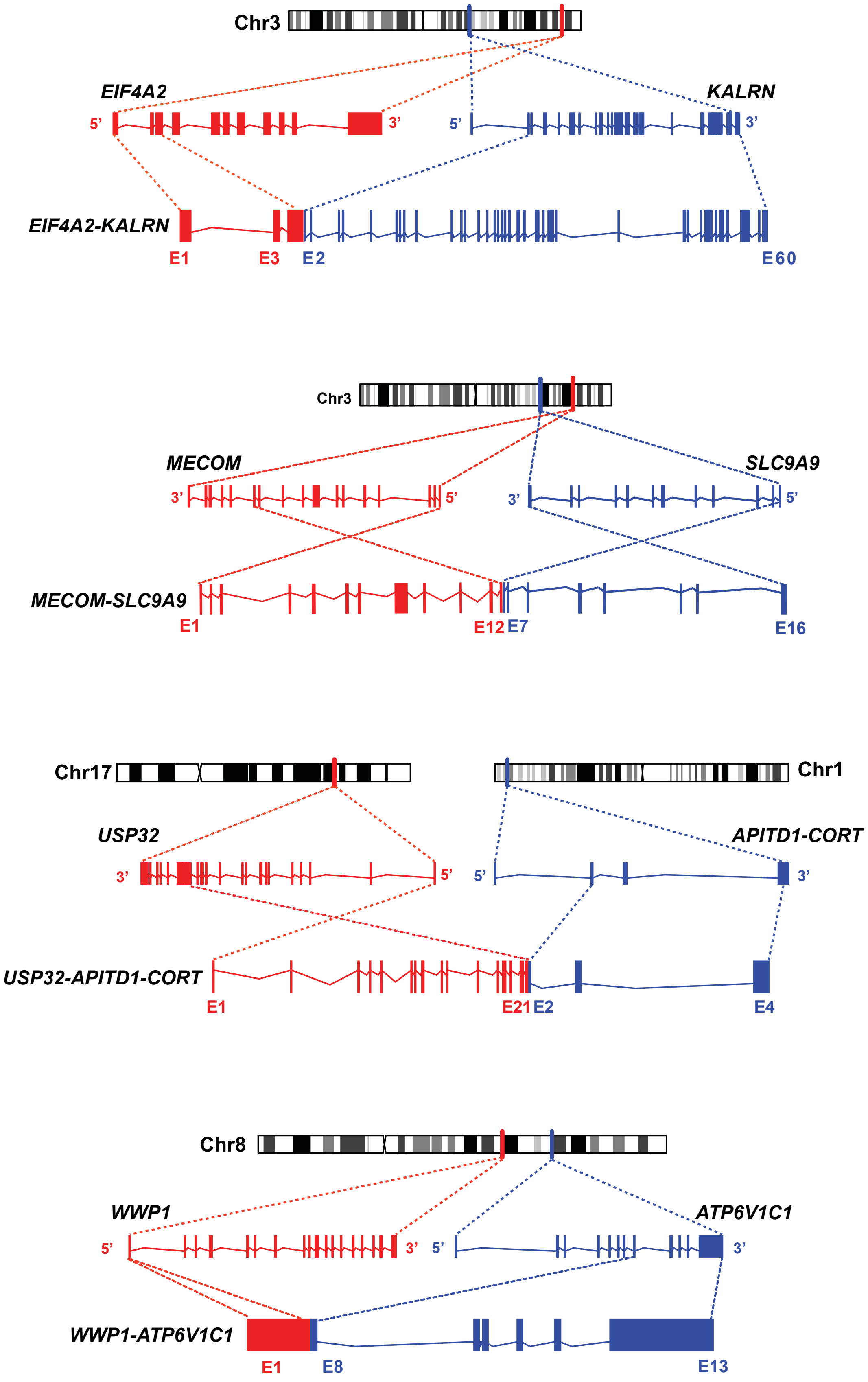

Chr3
EIF4A2
KALRN
5'
3'
5'
3'
EIF4A2-KALRN
E1
E3
E2
E60
Chr3
MECOM
SLC9A9
3'
5'
3'
5'
MECOM-SLC9A9
E1
E12
E7
E16
Chr17
Chr1
USP32
APITD1-CORT
3'
5'
5'
3'
USP32-APITD1-CORT
E1
E21
E2
E4
Chr8
WWP1
ATP6V1C1
5'
3'
5'
3'
WWP1-ATP6V1C1
E1
E8
E13

Supplementary figure 14

mRNA expression level of IQGAP3 in 56 primary lung adenocarcinomas, 24 metastatic tumors and corresponding adjacent normal tissues profiled from RNA-seq.

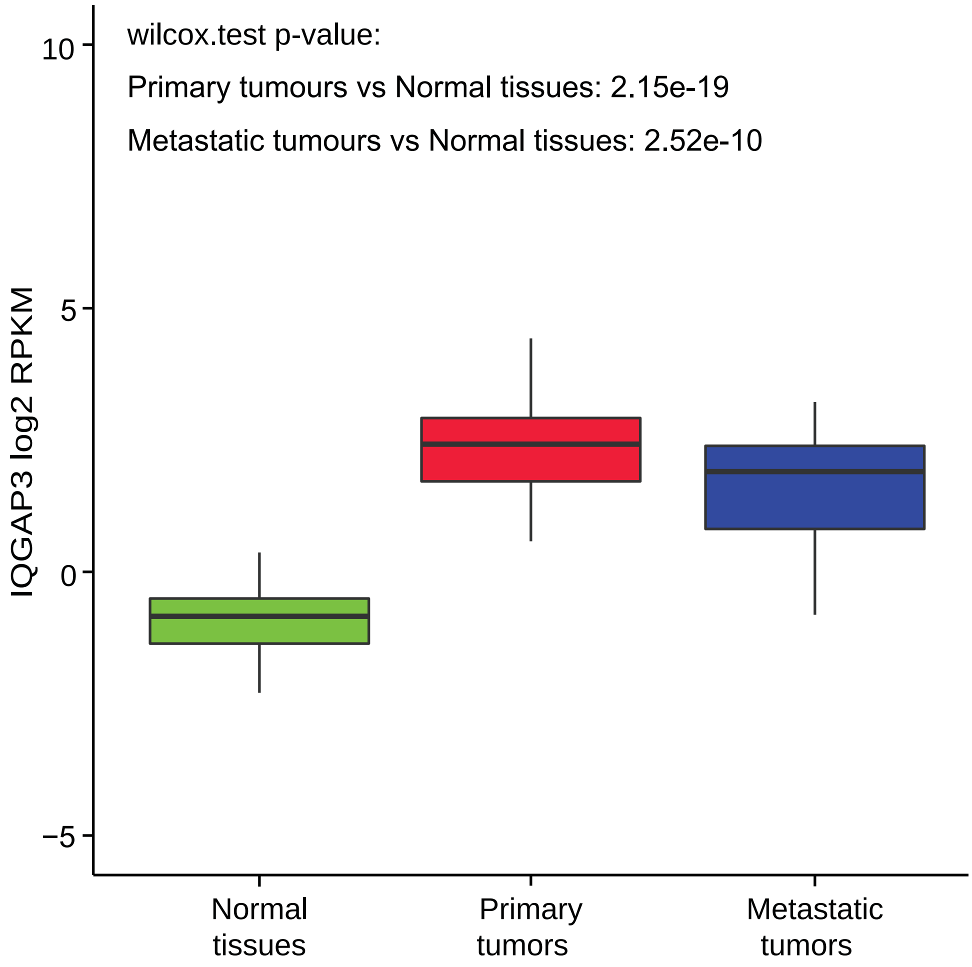

Supplementary figure 15

Immunohistochemistry staining indicated the higher expression level of IQGAP3 in tumors tissues of lung adenocarcinoma (left) than in normal tissues (right). Scale bars represent 100μm.

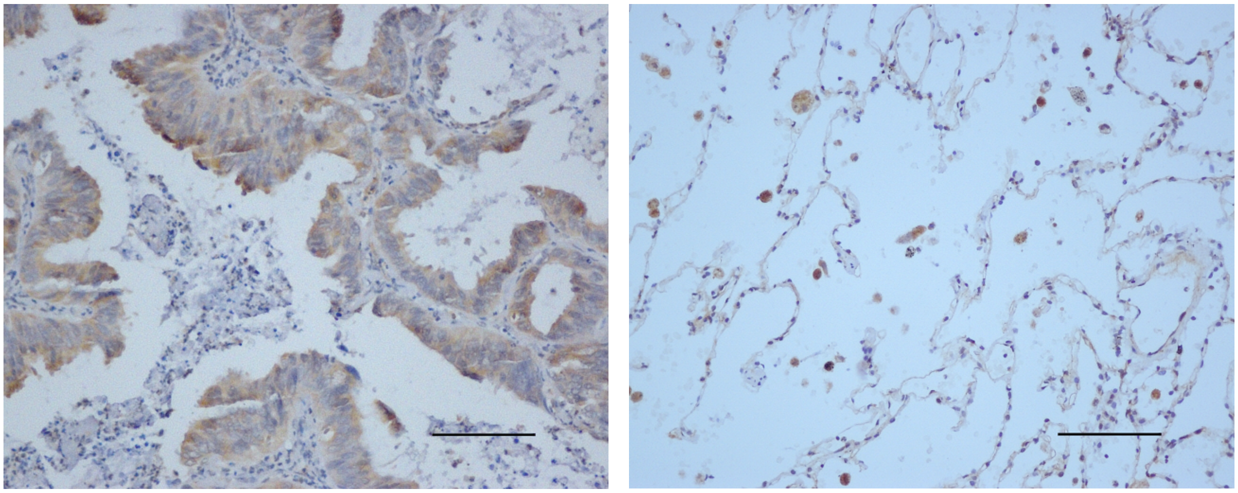

Supplementary figure 16

Kaplan-Meier survival curves shows that lung adenocarcinoma patients in validation cohort (n=71) with high expression level of IQGAP3 had significantly shorter overall survival and disease-free survival than those expressing low IQGAP3.

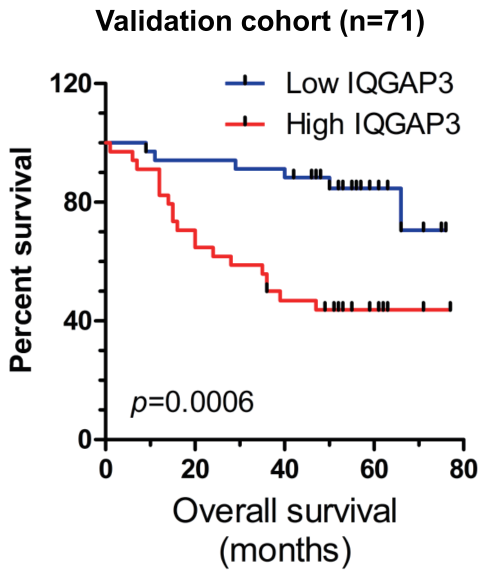

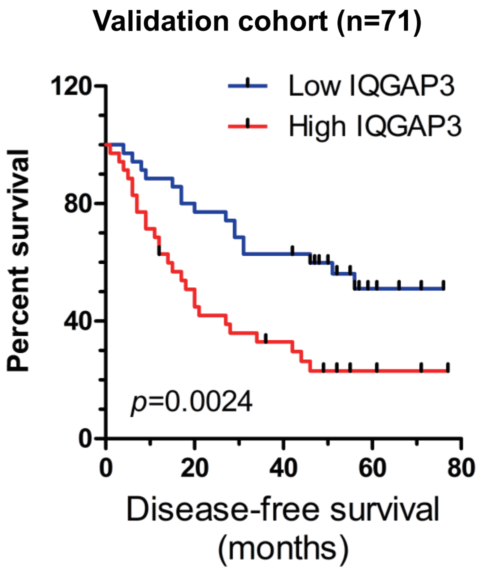

Supplementary figure 17

TMC6-IQGAP3 fusion indentified in case WG04. a) Schematic representation of TMC6-IQGAP3 fusion from whole genome and transicriptome sequencing, as well as Sanger sequencing chromatogram showing the validated breakpoint. b) Outlier high expression of IQGAP3 in WG04 primary and metastatic tumors. RPKM, reads per kilobase of exon region in a gene per million mapped reads. c) RNA-seq coverage analysis of TMC6 (top) and IQGAP3 (bottom) in the tumor and matched normal tissue from WG04. Two transcripts of TMC6 and one transcript of IQGAP3 are shown. Red lines indicate breakpoints. E, exon.

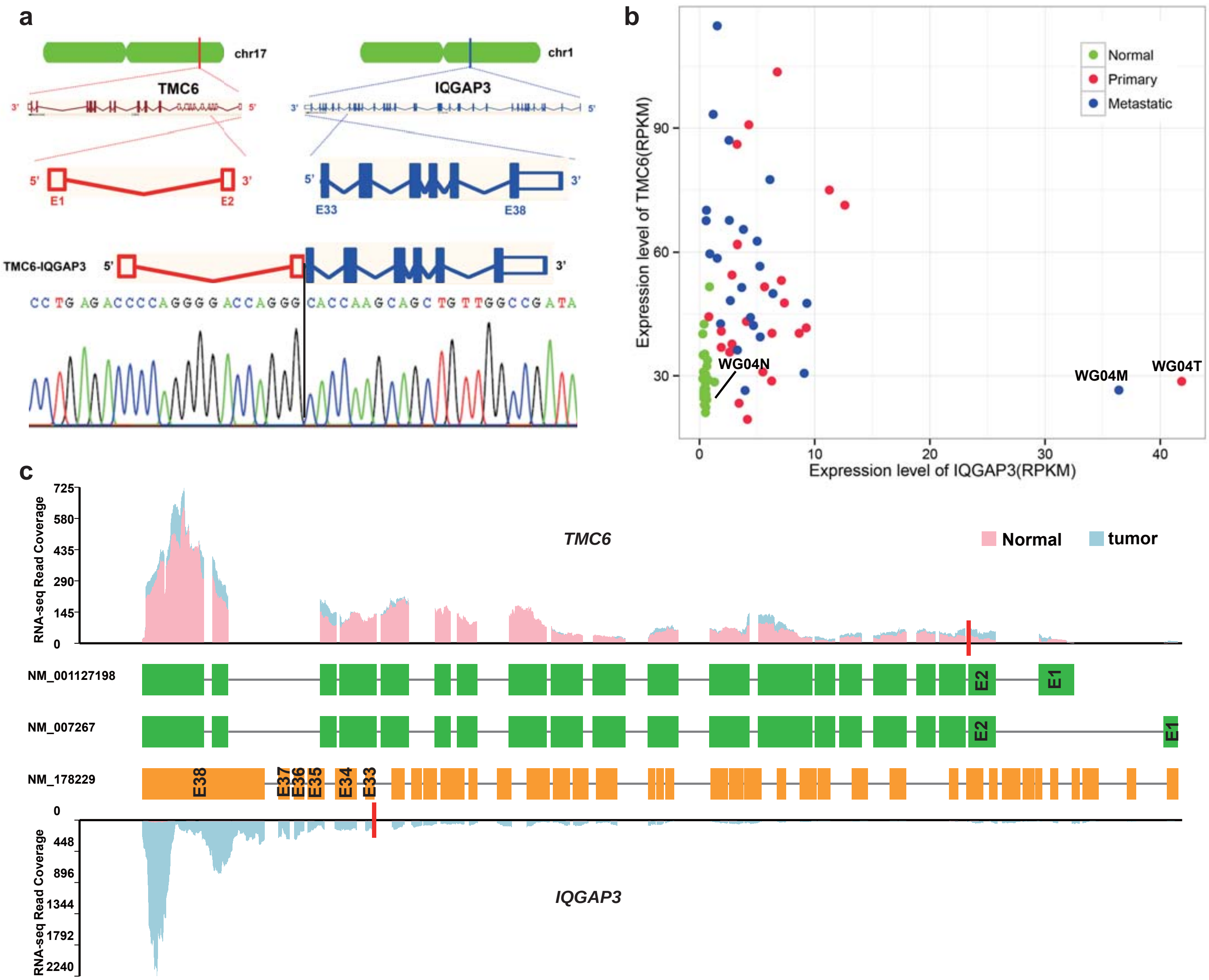

Supplement: Supplementary Information — Supplementary Figures 1-17 [file ncomms10131-s1.pdf]
